# Agent-based modelling of complex factors impacting malaria prevalence

**DOI:** 10.1186/s12936-021-03721-2

**Published:** 2021-04-15

**Authors:** Miracle Amadi, Anna Shcherbacheva, Heikki Haario

**Affiliations:** 1 LUT School of Engineering Science, Lappeenranta University of Technology, Yliopistonkatu 34, Lappeenranta, Finland; 2 Finnish Geospatial Research Institute, Geodeetinrinne 2, 02431 Masala, Finland; 3 Finnish Meteorological Institute, Erik Palménin aukio 1, 00560 Helsinki, Finland

**Keywords:** Computational biology, Socio-economic factors, Agent-based modelling, Prevention of reintroduction, Long-lasting insecticidal nets, Multiscale modelling

## Abstract

**Background:**

Increasingly complex models have been developed to characterize the transmission dynamics of malaria. The multiplicity of malaria transmission factors calls for a realistic modelling approach that incorporates various complex factors such as the effect of control measures, behavioural impacts of the parasites to the vector, or socio-economic variables. Indeed, the crucial impact of household size in eliminating malaria has been emphasized in previous studies. However, increasing complexity also increases the difficulty of calibrating model parameters. Moreover, despite the availability of much field data, a common pitfall in malaria transmission modelling is to obtain data that could be directly used for model calibration.

**Methods:**

In this work, an approach that provides a way to combine in situ field data with the parameters of malaria transmission models is presented. This is achieved by agent-based stochastic simulations, initially calibrated with hut-level experimental data. The simulation results provide synthetic data for regression analysis that enable the calibration of key parameters of classical models, such as biting rates and vector mortality. In lieu of developing complex dynamical models, the approach is demonstrated using most classical malaria models, but with the model parameters calibrated to account for such complex factors. The performance of the approach is tested against a wide range of field data for Entomological Inoculation Rate (EIR) values.

**Results:**

The overall transmission characteristics can be estimated by including various features that impact EIR and malaria incidence, for instance by reducing the mosquito–human contact rates and increasing the mortality through control measures or socio-economic factors.

**Conclusion:**

Complex phenomena such as the impact of the coverage of the population with long-lasting insecticidal nets (LLINs), changes in behaviour of the infected vector and the impact of socio-economic factors can be included in continuous level modelling. Though the present work should be interpreted as a proof of concept, based on one set of field data only, certain interesting conclusions can already be drawn. While the present work focuses on malaria, the computational approach is generic, and can be applied to other cases where suitable in situ data is available.

**Supplementary Information:**

The online version contains supplementary material available at 10.1186/s12936-021-03721-2.

## Background

Malaria is often regarded as a socio-economic disease associated with poverty and underdevelopment. The incidence of the disease tends to decline with economic development and associated improvement in domestic conditions, such as quality of housing and availability of medical aid [1, 2]. The elimination of malaria in, for instance, Finland was preconditioned on a drop in household size [2–4]. However, malaria imported by visitors and migrants carries the risk of re-introducing malaria in areas that have suitable vectors and climatic conditions [5]. For instance, the proportion of imported malaria cases due to migrants in Europe has recently increased from 14 to 83% [6–9]. It is therefore topical to reconsider various factors controlling the spread of malaria.

Classical compartmental models contain a limited account of the complex processes of malaria transmission dynamics, and more detailed models tend to get over-loaded with model parameters that are difficult to calibrate against real data [10]. Here, an approach to alleviate this dilemma is demonstrated by a combination of individual or agent-based modelling (ABM) strategy together with compartmental modelling. The ABM approach has become popular due to its enhanced realism, flexibility, explicitness and the advantages of spatial simulations with high resolution (see [11]). An agent-based modelling approach is employed in order to simulate the impact of factors such as intervention measures, household size, and the behavioural changes of the vector. The ABM results are then linked to basic dynamic transmission models in order to enable predictions on the level of public health [12–14].

The ABM modelling is done first for a single host in the hut, and then on a household level, with multiple individuals sleeping under the same roof. Subsequently, the household-level model is extended to community-level scenarios, enabling simulations of heterogeneity of mosquito-to-human contact rates due to partial coverage with nets or different household sizes. The crucial impact of socio-economic factors such as household size has been emphasized in [2–4]. The ABM simulations provide a ‘computational laboratory’ where data reflecting the impact of various complex factors can be produced. Upon repeated simulations, the ABM outputs can be used as synthetic data to produce regression models for the factors considered. Here, the focus is on household size, LLIN coverage, and alterations in mosquito behaviour induced by malaria parasite.

The agent-based model simulations are conducted over a ‘snapshot’ time period of one night. The results can be extended to continuous time by inserting the values fitted by the response surfaces as the key coefficients of classical compartmental models. Consequently, the impact of intervention measures or socioeconomic factors can be simulated over longer time periods, and to steady state. This allows for the estimation of the EIR [15] values in a wide variety of transmission scenarios.

**Fig. 1:**
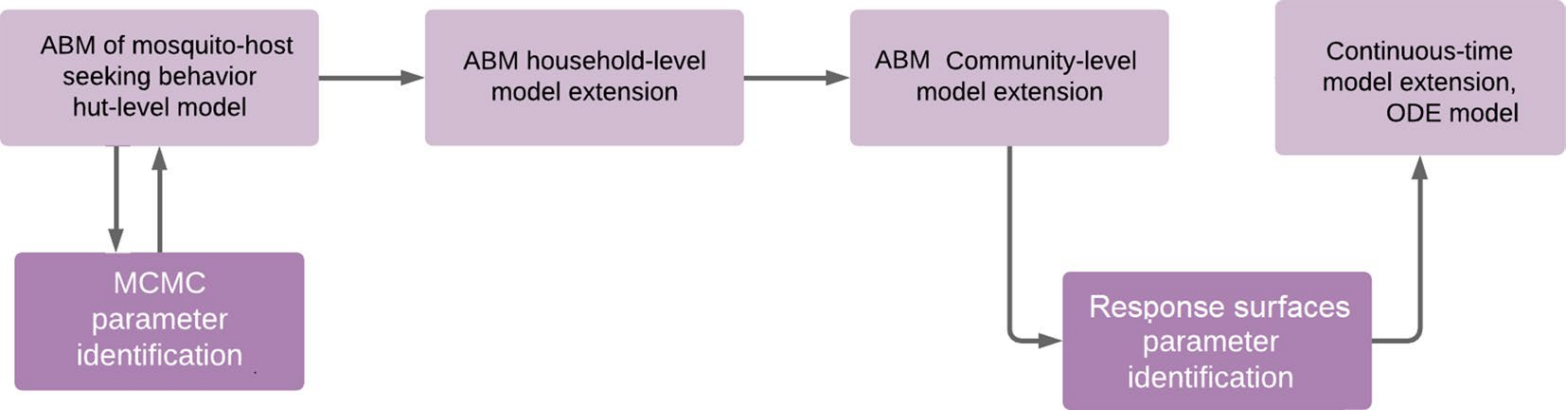
Schematic representation of transition from the ABM of mosquito host-seeking behaviour to the continuous modelling. The procedure is conducted separately for each of the mosquito species and the chemicals under study

The work-flow followed in the present study is summarized in the schematic illustration given in Fig. 1. The modelling process is iterative as there is back and forth movement from MCMC parameter identification to ABM of mosquito host-seeking behavior, such that the model fits the data well.

**Fig. 2:**
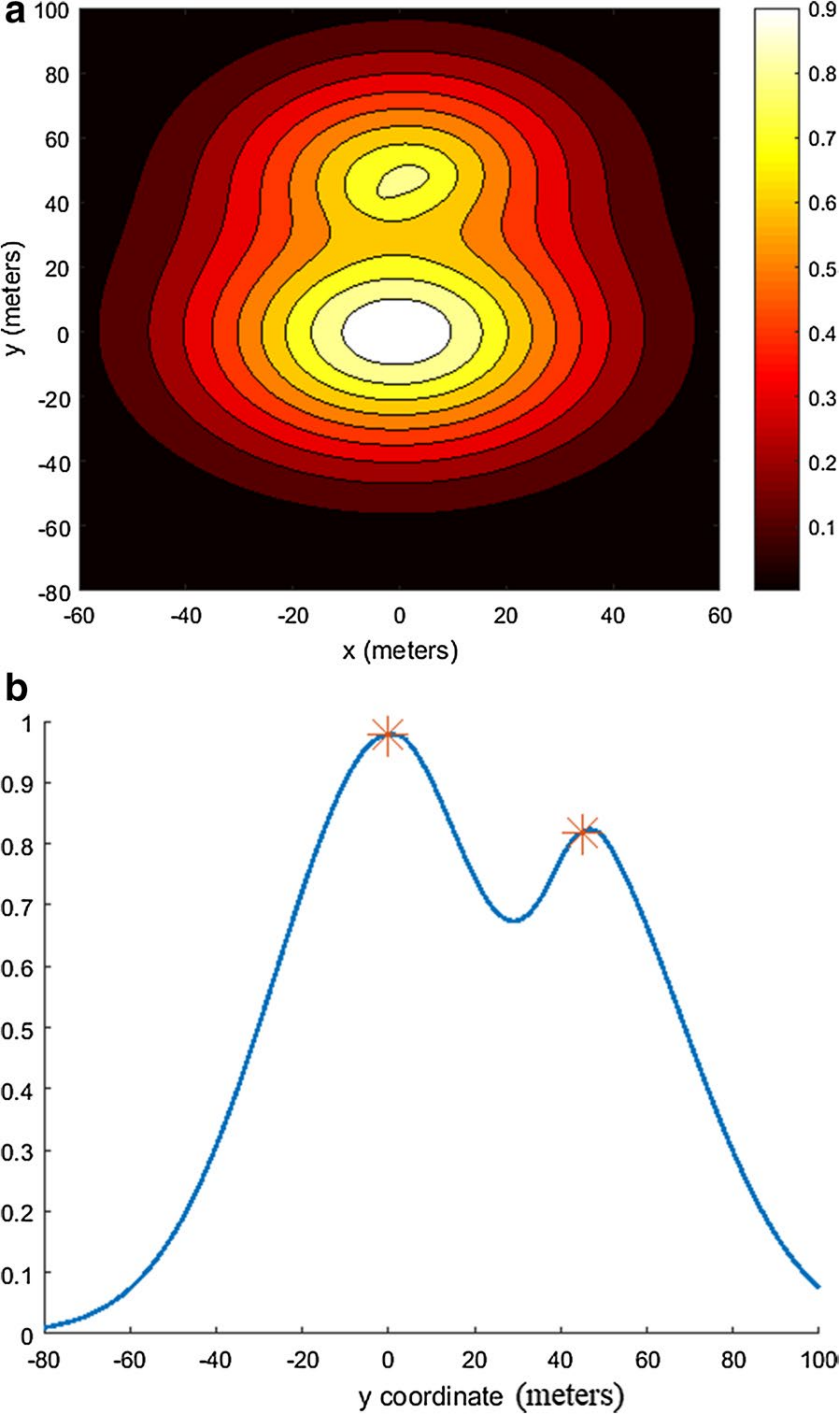
Softmax function in a special case of two households. The first household includes six individuals (located (0,0)) and the other household consists of two individuals (located at (0,45)) for $d_{50} = 10,s = 5$** a** 2D plot,** b** 1D plot along the y axis

Other studies have also estimated key parameter statistics from data on experimental hut trials and subsequently employed them in dynamic transmission models to enable public predictions. In the work by Churcher et al. [12], key parameters of the continuous model [16] were estimated using statistical models (such as binomial and mixed effect models) and calibrated with hut-level experiment data. Sherrard-Smith et al. [17] systematically assessed experimental hut data to characterize different indoor residual spray (IRS) product efficacies in terms of mosquito mortality, blood-feeding inhibition and deterrence against *Anopheles* mosquitoes, when fitted with statistical models. The impacts of IRS assessed from experimental hut trials are extrapolated for public health predictions in areas with different levels of coverage and pyrethroid resistance using the mathematical model of malaria transmission from [16]. Using a slightly different approach, Okumu et al. [13] directly inputted values of relevant parameters from experimental hut trials into their transmission model to make public predictions. This model additionally considered animal hosts (cattle) and predicted community-level impact on malaria transmission at high coverage (80%) using direct data from hut-level trials for various combinations of untreated nets or LLINs with IRS. Another related study [18] considered how coverage with ITNs (from 0 to 100%) influence the intensity of malaria transmission using an elaborated description of the classic feeding cycle model. The approach in this study differs from the above papers as it presents the model of mosquito host-seeking behaviour in a hut in terms of the mosquitoes’ attraction to the host, host-seeking orientation, biting and death rate. The agent-based simulation of mosquito behavior at hut-level in the presence of different insecticides is then calibrated with field data from [19]. The ABM approach enables modelling of behavioral changes typical for infected mosquitoes at the household level and subsequent extension to community-level simulations using households of different sizes. Thus, upon simulating the ABM, the key ODE model parameters are created, unlike in [18] which is model-based and parameter values are mainly assumed. Additionally, the LLIN coverage and household size are elaborately considered to range from 0 to 100%, and 2 to 10 respectively. This approach enables integration of socio-economic factors and the study of malaria prevalence in a population at varied protection levels, while in [12, 13] a certain level of coverage is assumed.

The rest of the paper is organized as follows. In "Methods" section, the basic agent-based modelling approach at the hut level with a single host and its extension are presented. Next, the ABM extension to household and and subsequently to community level are described. Then, the next subsection discusses the regression applied to the outputs of community-level simulations. The extension of the response surface results to continuous time is given in "Results" section. Finally, the discussion is presented in the last section.

## Methods

### Basic ABM host-seeking model with a single host

A previous work [20] presented an ABM simulation approach for mosquito host-seeking behaviour on hut-level in the presence of LLIN, calibrated for one case of the treatment data from [19]. Here, the model is extended in several ways to make it capable of reproducing the data of other insecticidal treatments, and to enable the extension to continuous time modelling done in "Results" section. The basic modelling approaches utilized in [20] is briefly recalled and the modifications made in the present work is pinpointed. The model developed in this study consists of four basic components, where each of the components features a number of associated attributes, see the summary in Table 1. Additionally, the properties that are assigned individually for each of the mosquito agents and updated within the simulation (see Table 2) are listed. The model components and the property list of mosquitoes are described in detail in this subsection.

**Table 1:** Model components

Model component	Attributes	Definition
Host-seeking	CO$_2$ concentration, Klinotaxis	Equation 2
	Distance-dependent attraction	Equation 5
	Host seeking time	
Motion	Random walk, accept/reject steps	Equation3
	Excito-repellency	Equation 11
Poisoning	Accumulation of the chemical dosage	Equation 7
	Detoxification	Equation 12
Death	Natural mortality	Equation 6
	Insecticide-induced mortality	Equation 8
	Delayed mortality	Equation 6 with model extension

#### Motion and host-seeking

The mosquito attraction model is based on the assumption that a mosquito estimates the direction of odour increase (the gradient) by the mechanism of klinotaxis [21]. During this plume-tracking behaviour, the mosquito samples the host odour at one location, changes location and then repeats the sampling, and uses its memory of the concentrations previously encountered to choose the next position [22, 23]. Imitating this process, the flight of mosquitoes is modelled as a discrete-time correlated random walk. Suppose that a mosquito agent is at position $\mathbf {x}^{n-1}$ at time step $n-1$. A new position $\mathbf {x}^n$ is selected by:

1 $$\begin{aligned} \mathbf {x}^n=\mathbf {x}^{n-1}+\delta \mathbf {W}, \end{aligned}$$

where the increment $\delta \mathbf {W}$ added to $\mathbf {x}^{n-1}$ is sampled in random direction, with a step size given by a normal distribution $N(\mathbf {x}_0,\sigma ^2 I)$. In the experimental runs, the parameters $\mathbf {x}_\mathbf {0}, \sigma$ were matched to imitate the real flight speed of a mosquito [20]. Mosquitoes are able to identify the host by making use of the olfactory cues that are given off by the host. As a primary stimuli, they move in response to the carbon dioxide ($\hbox {CO}_{2}$) exhaled by vertebrates. Additionally, at a short distance to the host, mosquitoes are able to discern by vision, olfaction and by using the heat sensors located around their mouthparts. In general, mosquitoes are unable to detect human prey from a distance greater than 80 m [22]. The concentration of attractive odour emitted from an individual host is modelled as a Gaussian kernel centered at a spatial position of the host $\mathbf {x}^h$:

2 $$\begin{aligned} C(\mathbf {x},\mathbf {x}^\mathbf {h})=\exp \left[ -\frac{d^2(\mathbf {x}, \mathbf {x}^h)}{2\sigma ^2_a}\right] , \end{aligned}$$

where $\mathbf {x}$ denotes the position of the mosquito, and *C* stands for the concentration that enables a mosquito to sense the host at a distance $d(\mathbf {x},\mathbf {x}^h)$. Note that the impact of wind is omitted for simplicity. The standard deviation of the Gaussian $\sigma _a$ determines a maximal distance at which the mosquito is able to sense the host.

The mosquito flight is given by the above random walk in the absence of attraction effects towards the host. However, when there are attraction effects, the main features of the Metropolis algorithm is employed in order to simulate the random walk directionally biased by attraction [24]. Suppose that a mosquito takes a step from point $\mathbf {x}^{n-1}$ to a candidate point $\mathbf {x}^n$ with respective function values as $p_{n-1}$ and $p_n$. Then a new point is accepted with probability:

3 $$\begin{aligned} \alpha _a(\mathbf {x}^\mathbf {n}|\mathbf {x}^{\mathbf {n-1}}) =\min \left( 1,\frac{p(\mathbf {x}^{\mathbf {n}})}{p(\mathbf {x}^{\mathbf {n-1}})}\right) , \end{aligned}$$

where $p(\mathbf {x}^{\mathbf {n}})/p(\mathbf {x}^{\mathbf {n-1}})$ is the ratio of the attraction potential function $p(\mathbf {x})$ defined at each point $\mathbf {x}$, which depends on the concentration and other attraction factors. In order to parsimoniously account for other short-distance attraction factors, the attraction potential function is defined as:

4 $$\begin{aligned} p(\mathbf {x})=\exp \left( C(\mathbf {x})/\sigma _{acc}\right) \end{aligned}$$

with a scaling factor $\sigma _{acc}$ that depends on the distance to the host. Outside the plume $p(\mathbf {x}) = 1$, so by Eq. 3 all steps are accepted, while closer to the host steps away from the host are increasingly rejected due to activation of the heat sensors. At a short distance to the host this is modelled by a linear scaling factor as:

5 $$\begin{aligned} \sigma _{acc}(\mathbf {x},\mathbf {x}^h) =\left\{ \begin{array}{lr} \sigma ^1_{acc}+\sigma ^2_{acc}d(\mathbf {x},\mathbf {x}^h),&{} d(\mathbf {x},\mathbf {x}^h) \le 80 \\ \sigma ^{\max }_{acc},&{} d(\mathbf {x},\mathbf {x}^h) > 80. \end{array}\right. \end{aligned}$$

The above function increases from the minimum value of $\sigma ^1_{acc}$ with a slope given by the parameter $\sigma ^2_{acc}$ until it is replaced by a constant which suitably provides a purely random movement outside the concentration plume [20].

#### Death, poisoning and repellency

The LLINs are assumed to be equipped with repellent and poisoning effects. In the absence of chemical treatment, the total probability of death reduces to the natural mortality rate. The continuous-time mortality rate $\mu$ can be transformed into a probability of death per unit time $\Delta t$ by:

6 $$\begin{aligned} \alpha ^{\Delta t}=\min \big \{1,\mu \Delta t\big \}, \end{aligned}$$

where $\Delta t = 2$ s is used for all simulations, and a value for $\mu$ taken from the literature (see [20] for more details). This conforms with the 34-h natural mortality rates reported for *Anopheles gambiae* and *Anopheles arabiensis* as 10%, see [25] .

The poisoning effect is modelled with the assumption that at a time instance *i*, mosquito consumes a dosage of chemical $D_i$ spread on the treated net upon contact to the net surface. Thus, the total accumulated dosage $C_{tot}$ is computed as the number of contacts with the net:

7 $$\begin{aligned} C_{tot}(n+1) = \sum \limits _{i=1}^{n+1} D_i =C_{tot}(n) + D_{n+1}, \end{aligned}$$

where $D_i$ is non-zero in case of hitting the net surface (i.e., equal to the unit dosage), and zero otherwise.

The insecticidal-induced increase in mortality is then modelled as:

8 $$\begin{aligned} \alpha ^{\Delta t}_p(n) =\mu _p C_{tot}(n)\Delta t, \end{aligned}$$

where the effective poisoning impact is obtained by a scaling coefficient $\mu _p$ which depends on the given insecticide used for LLIN treatment.

So the total probability of death per unit change in time $\Delta t$ is modelled as the sum of natural and insecticide-induced mortality:

9 $$\begin{aligned} \alpha _{death}=\min \{1,\alpha ^{\Delta t}+\alpha ^{\Delta t}_p (n)\}. \end{aligned}$$

Repellency is modelled with the logistic curve multiplied with the repulsion intensity parameter *r*:

10 $$\begin{aligned} C_{rej}= r \left[ 1-1/\Big (1+\exp \big (-\left( d(\mathbf {x},\mathbf {x}^h)-d_{50} \right) /s\big )\Big )\right] , \end{aligned}$$

where $d(\mathbf {x},\mathbf {x}^h)$ denotes the distance from the mosquito to the protected human and *r* ranges from 0 to 1. The parameters $d_{50}$ and *s* determine the range of coverage and the spread of the chemical. The logistic function is modified such that the rejection probability at the candidate position $\mathbf {x}$ amplifies as the mosquito approaches the source of repellent. Considering the properties of modern insecticidal treatments [26], the spatial range of the repellent *s* is taken to be small such that the impact is only within the vicinity of the net.

The repulsion by LLIN is computed in two stages. First, the accept/reject step is applied, where the probability of rejection is given by a logistic function describing the contact irritancy caused by the chemical, as given in Eq. 10. Next, the physical net barrier is taken into account, for which the probability of being blocked by the net is assigned as $p_{net}<1$ such that there is a non-zero chance for penetration.

### Model extensions

#### Motion and host-seeking: excito-repellency

The aim is to keep the host-seeking model as minimalistic as possible, by including only the indispensable factors listed in Table 1. It turns out, however, that the impact of different chemicals could not be fitted by the basic formulation given above. For instance, the model has to reproduce cases of higher exit and lower contact rates along with more than twice higher mortality rate for *An. gambiae* than *An. arabiensis*, following the data reported in [19]. Three new features necessary to characterize the impact of different chemicals on mosquitoes: metabolic detoxification [27, 28], delayed impact [19] and excito-repellency (or insecticide-induced exiting) [19, 29], are introduced. In order to account for insecticide-induced exiting, a scaling factor which not only depends on distance but also on repellent effect is further obtained. The inclusion of both distance and repellent effect is essential in order to properly fit the exit rates, as it accounts for generally higher exit rate when confronted with the treated nets as compared to the control case with the untreated nets. Thus, an excito-repellency parameter [29], $\mu _e$ is introduced, which depends on the mosquito species and the insecticide utilized in treating a given LLIN, parameterized as:

11 $$\begin{aligned} \sigma _{acc}(\mathbf {x},C_{tot})=\sigma _{acc}(\mathbf {x})+ \mu _e \cdot C_{tot}, \end{aligned}$$

where $C_{tot}$ denotes the total dosage of chemical consumed by the mosquito (see Eq. 7).

The other two included features: metabolic detoxification (see Eq. 12) and delayed mortality are explained next.

**Fig. 3:**
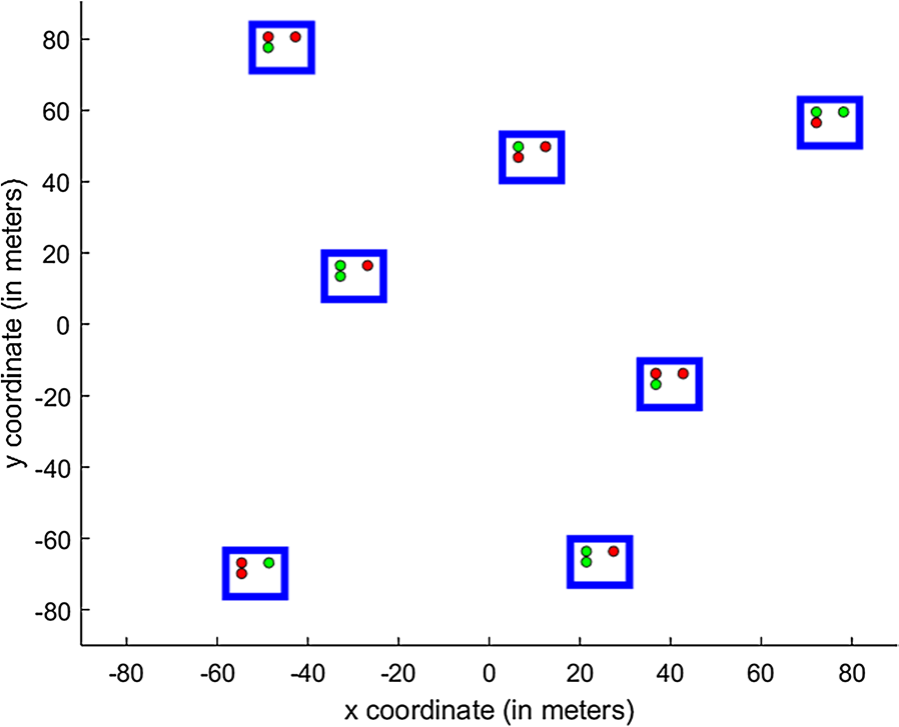
Randomly generated experimental layout with household size of three individuals. Here blue rectangles denote the houses, green/red circles mark individuals protected/non-protected with the impregnated nets

**Fig. 4:**
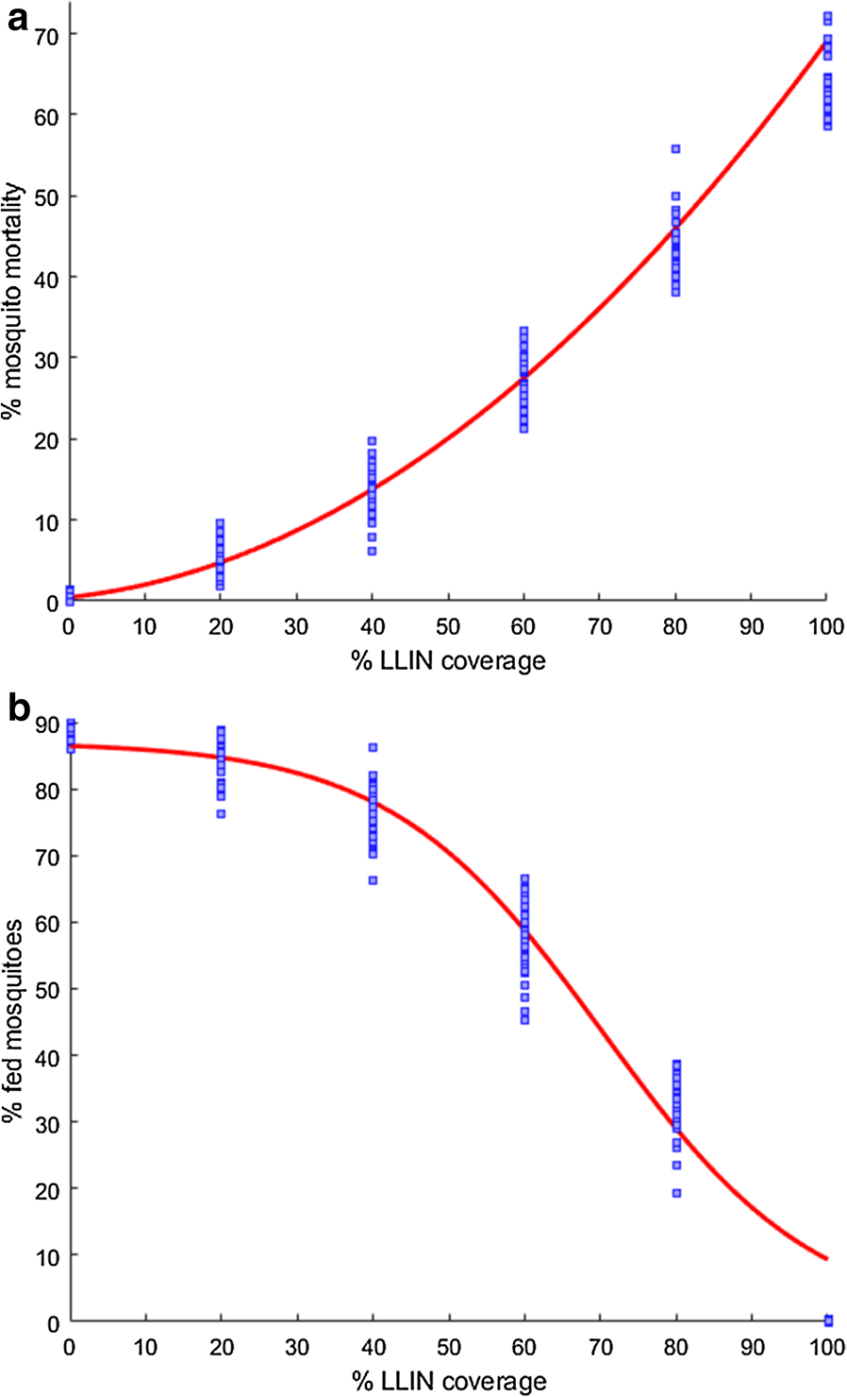
Uncertainty from the sampled parameters at hut level together with the variability of the community-level assumed parameters for **a** mortality rate,** b** fed rate, of *An. gambiae* when confronted with LLIN impregnated with an Alphacypermethrin treatment kit, fitted with respect to partial coverage of LLIN for the household size of 2 when assuming no behavioural alterations caused by the parasite

#### Poisoning and death: detoxification and delayed death rate

Here, the scenarios in the datasets from [19], where *An. arabiensis* is revealed to have consistently higher (or equal) feeding rate than *An. gambiae* but considerably lower death rate, are accounted for. These scenarios are inconsistent with the mechanism of the model presented in [20]. The inconsistency is explained by the fact that it is not possible to have simultaneously high feeding rate and low mortality rate if both the probability of death and that of successful feeding is proportional only to the number of contacts with the net. A number of probable reasons can be offered to account for the conflicting situation. One explanation is that the rate of poisoning is different for the two species because it takes time for the poison to get from the salivary glands to the neural system of mosquito and this time delay is suspected to be different for the two mosquito species. However, a large dosage is equally lethal for both *An. gambiae* and *An. arabiensis* and mosquitoes do not acquire the lethal dosage upon a single contact with the net but rather a sub-lethal dosage [19]. So, the explanation of detoxification is followed such that the chemical concentration is exponentially decaying with a rate $\alpha$ which depends on the chemical and mosquito species [28, 30]. Hence, given the previous dosage of the chemical $C_{tot}(n)$ at the step *n*, the dosage at the next step $n+1$ is calculated by modifying Eq. 7 as:

12 $$\begin{aligned} C_{tot}(n+1) =C_{tot}(n) + D_{n+1} -\alpha C_{tot}(n) \Delta t. \end{aligned}$$

Additionally, the delayed mortality that is a result of the prolonged impact of poison in mosquitoes is considered. Since poisoning effect is primarily associated with contact with the treated surface, some time is needed for the chemicals to penetrate and reach their target, which in turn depends on the physiological characteristics of the mosquito, such as the sensitivity of target proteins and the thickness of the cuticle [27]. Also, due to enzymatic detoxification, the knock-down time is prolonged. Owing to the high exit rates reported in [19], it was concluded that the mortality induced by the insecticides occurred only after a delay. Although the mosquitoes respond differently with different chemicals, the detailed modelling is spared and the enhanced probability of death is simply taken into account only after a 24-h time period as given by Eq. 6, with $\Delta t = 24\cdot 1800$.

The improved model of the chemical-induced exiting and mortality introduced can be calibrated for all the different treatment kits data from [19] (see Additional file 1 for a summary of the datasets). The model is capable of reproducing, e.g., the experimentally recorded lower contact rates along with more than twice higher mortality rates for *An. gambiae* as compared to *An. arabiensis* [19]. The calibration is performed using Bayesian sampling methods (adaptive MCMC) in the same way as in [20], more the details are given in Additional file 1. The motive of the MCMC simulations is to find the posterior distibutions of model parameters, that is ‘all’ parameter combinations that reproduce the measured data, within the accuracy given by the estimated error bounds of the data. While most of the parameters are reasonably well identified, some of them are clearly correlated. For instance, as the chemically enhanced mortality rates are now explained by both detoxification and exito-repellency, the respective parameters are strongly correlated with $\mu _p$, the earlier introduced death rate coefficient.

### Household-scale simulations: household size effect and behavioral alterations

Here, the description of the household and community level modelling is presented, adding more details to the preliminary demonstration given in [20]. First, the ABM of mosquito host-seeking behaviour is extended to the household level with multiple individuals sleeping under the same roof. Next, the modelling is extended to community-level scenarios with several households located in the landscape of interest. See the illustration of the workflow in Fig. 1.

**Fig. 5:**
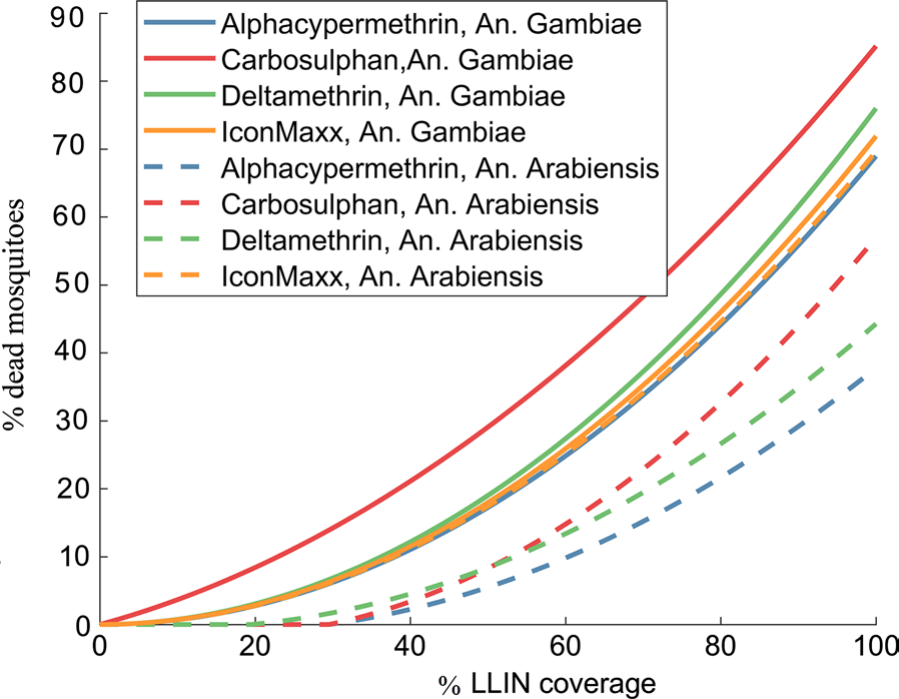
Mortality rates regression models. *An. gambiae* (solid lines) and *An. arabiensis* (dashed lines) fitted with respect to partial coverage of LLIN (pLLIN) for four LLIN treatment kits: Alphacypermethrin (blue) Carbosulphan (red), Deltamethrin (magenta) and IconMaxx LN (black)

**Fig. 6:**
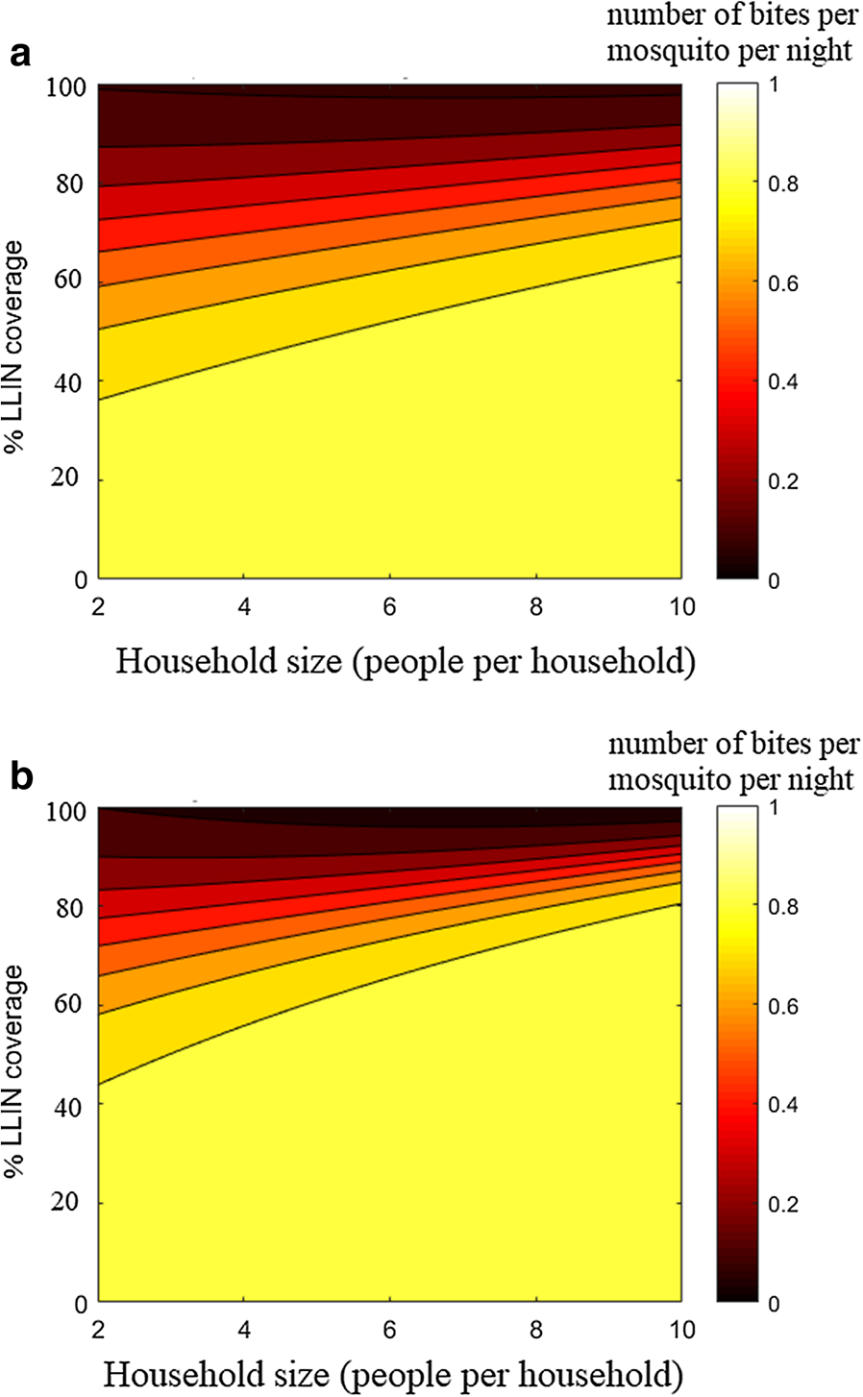
Contact rates.** a** *An. gambiae* and** b** *An. arabiensis* fitted with respect to household size and partial coverage of LLIN when confronted with LLIN impregnated with an Alphacypermethrin treatment kit, assuming no behavioural alterations caused by the parasite

A significant correlation between malaria reduction and the decline in typical household size in malaria-endemic countries is discussed in [2–4]. It was concluded that the larger the number of people sleeping together in non-segregated quarters, the higher the probability of transmitting the infection to new uninfected humans [2]. In Finland, for instance, the probability of malaria disappearance increased when the average number of individuals in one household declined below the threshold of four people, even when no specific control measures were applied [2, 4].

Naturally, there are several other household-related factors that can influence the rate of transmission apart from the household size. Such factors include, e.g., household practices like livestock/poultry rearing, as well as the rate of hygiene maintenance in a given household [31]. For simplicity, these factors are omitted here. The interest of the ABM simulations is in the impact of LLINs. The mosquito density *m* which can be impacted by these omitted factors, is taken into account in the ODE model. Also, the situation is restricted to a given number of persons sleeping together in the same room, while the approach can be extended also to cases of many people sleeping in separated quarters. The aim here is to demonstrate how household-level factors can be included in ABM simulations, and how even most rudimentary considerations impact the modelling outcomes.

On entering a household, the mosquito is assumed to choose one of the hosts randomly. After this, the modelling reduces to the previous case of a single host in the hut. A few changes are needed, however. The tendency of mosquitoes to switch to neighbouring individuals after spending a certain time in unsuccessful attempts to feed on a protected host, should be considered. Thus, an additional parameter, $t_{max}^{host}$, the maximal time spent while attempting to feed on a protected host, is introduced [32, 33]. In the absence of more specific knowledge, the parameter is set to 10 min. In addition, same as in the hut-level experiment, mosquitoes are restricted to a maximum host-seeking time, $t_{max}$ inside the household after which they switch to a random walk with no influence of the human bait. Another difference is easier exit from a usual household compared to that from the special design of experimental huts. A typical human dwelling [34] is mimicked by setting the probability of exit to constant value that produces about 90% exit rates per night in the absence of chemical treatment.

Infection with malaria parasites has been shown to alter the behaviour of mosquitoes, with varying effects that are based on the life stage of the parasite [35]. The underlying mechanisms that engender these behavioural alterations are not fully explored but mostly result from at least two manipulation processes. Firstly, the parasite increases the mosquito’s motivation to continue a meal after interruption, thus increasing its probability of taking several bites. Secondly, the parasite impairs the vector’s ability to obtain a full blood meal upon a single bite, inducing the vector to bite several times before it is fully engorged [36, 37]. These behavioural changes associated with infection seem likely to be an evolutionary mechanism that has been developed by malaria parasites, which enhances the spread of infection [38, 39]. A more profound understanding of the behavioural tendencies of parasite-infected mosquitoes alongside the stage-specific changes in their host-seeking behaviour could provide a potential target for genetic manipulation of mosquitoes, as a preventive measure for the elimination of malaria infection [40].

In the simulation, the impact of multiple biting typical for infected mosquitoes is accounted for. Both infected and uninfected mosquitoes are assumed to have the tendency of feeding on multiple hosts [41]. However the tendency of multiple feeding is higher for infected mosquitoes. Thus, the statistics from [36] is employed, which indicate that 10% of uninfected and 22% of infected mosquitoes obtain a blood meal on at least two hosts, while assuming that the maximal number of successful feeding attempts can be up to 5 for both, depending on the accessibility of the hosts. The dosage of blood sufficient for ovipositing is assumed to be achieved after the maximal number of successful feeding attempts is reached. Note that the hut-level data, with one person in the hut, does not contain information on the alterations in behaviour during the host-seeking, so at this point, the literature is relied on. On the other hand, the conjecture that humans infected by the parasite attract more mosquitoes [42] is not included in the simulations, since the hypothesized enhanced attractiveness has demonstrated insignificant impact on the outcome of the simulations (see [43]).

Note that in the simulations, the model parameter values are also re-sampled from the estimated parameter posteriors at each successive iteration of the algorithm to account for parameter uncertainty (see Additional file 1). The main model parameters are summarized in Table 3.

**Table 2:** Property list of each agent and the relevant model component

Property index	Property	Model component
1	Spatial position	Motion
2	Inside/outside the hut	Motion
3	Inside/outside the net	Motion
4	Trapped	Motion
5	$CO_2$ concentration	Motion
6	Fed	Host-seeking
7	Time indoors	Host-seeking
8	Klinotaxis	Host-seeking
9	Dead	Death (Poisoning)
10	Accumulated dosage of chemical	Poisoning

### Community-scale simulations

Next, the modelling is extended to a community-scale experiment with the primary aim of quantifying the effect of household size and a partial population coverage with LLINs (see Fig. 1). Similar to the hut-level case, the movement of mosquitoes in the odour plume is governed by the mechanism of klinotaxis, but the concentration which enables the mosquitoes to sense the hosts is now computed as a function of a weighted sum of distances from all the individual hosts:

13 $$\begin{aligned} C_a^{tot}(\mathbf {x}) = C(W_n, \mathbf {x},\mathbf {x}^h_n)=\exp \left[ -\left( \frac{\sum \limits _{n = 1}^{N_{h}}W_nd(\mathbf {x},\mathbf {x}^h_n)}{\sqrt{2}\sigma _a}\right) ^2\right] . \end{aligned}$$

Here $N_{h}$ denotes the total number of individuals in the community, $d(\mathbf {x},\mathbf {x}^h_n)$ stands for the distance from mosquito position $\mathbf {x}$ to the host location $\mathbf {x}^h_n$, and $W_n$ is the weight attributed to the host *n*.

The total attracting concentration is modelled following the idea of the *softmax* function, which has been widely adopted in machine learning and neural networks (see [44, 45]). The weight $W_n$ is introduced to account for the fact that a mosquito’s response to the cue emitted from the households increases at a short distance of 5–15 m, depending on the mosquito species, due to their attraction to visually conspicuous objects [46–48]. Here, the main focus is placed on the nearest target concept, which practically means that at a short distance factors other than just the $CO_2$ alone also cause the mosquito to localize the search, as reported in [46–48]. Following this reasoning, the non-normalized weights $\hat{W_n}$ are introduced inversely proportional to the distance:

14 $$\begin{aligned} \hat{W}_n(\mathbf {x},\mathbf {x}^h_n) = (1 - 1/\exp (-(d(\mathbf {x},\mathbf {x}^{h}_{n}) - d_{50}^h)/s^h), \quad \mathbf {x}^h_n \in \mathbf {x}^{h}_{n}. \end{aligned}$$

The value of $d_{50}^h$ is set to be 10 m, to conform with the conjecture that at a distance of less than 10 m from the households, within which a mosquito is able to discern shapes, the concentration sensed by the mosquito is assumed to be that which is emitted from the closest household only. The second parameter $s^h > 0$ governs the spatial range of sensitivity that enhances at a short distance. Here, the value $s^h = 5$ m is used to account for the gradual boost of the mosquito’s response to the cues. The weights $\hat{W_n}(\mathbf {x},\mathbf {x}^h_n)$ are normalized by $W_n =\hat{W_n}/\sum ^{N_h}_{j = 1} \hat{W_j}$.

Note that the form of Eq. 13 is consistent with the evidence that larger agglomerates emit stronger odours, hence, attracting more mosquitoes [23] (see the illustration in Fig. 2).

**Fig. 7:**
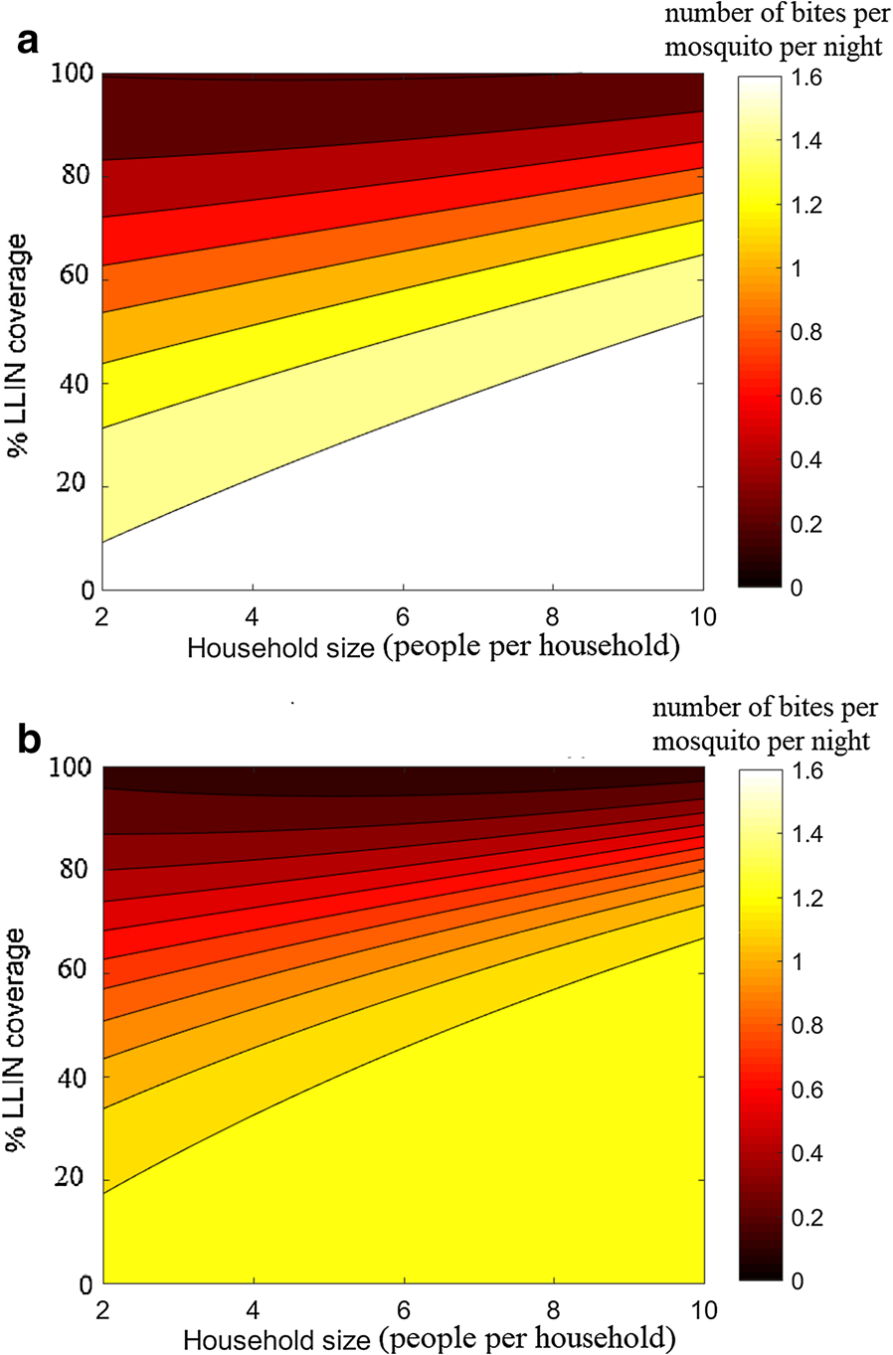
Contact rates.** a** Infected and** b** uninfected *An. arabiensis* when confronted with LLIN impregnated with an Alphacypermethrin treatment kit, fitted with respect to household size and partial coverage of LLIN (pLLIN) when assuming behavioural alterations by parasite

**Fig. 8:**
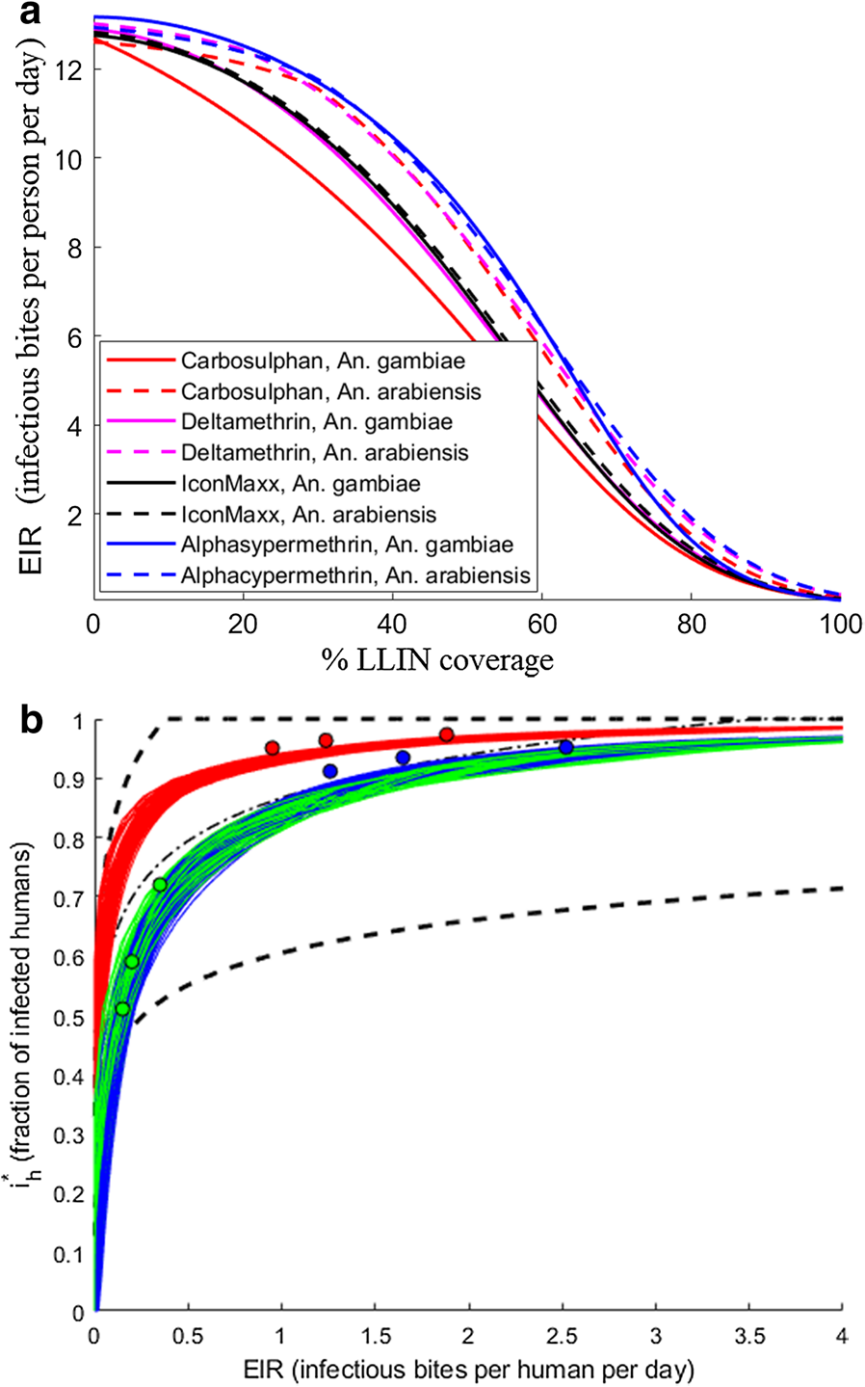
Equilibrium EIR values, assuming behavioural alterations for household size 4.** a** Equilibrium EIR values as continuous functions of LLIN coverage.** b** Equilibrium values of malaria prevalence $i_h^*$ versus EIR, in comparison to the mean trend (black dash-dotted line) and 95% confidence bounds (black dashed lines) as given in [50]. High (red circles), medium (blue circles) and low (green circles) transmission settings from [10], together with simulated values

Environmental factors such as wind and intermittency of the plume are omitted for simplicity. Initially in the simulations, mosquitoes are randomly placed inside the simulated transmission domain of 25,600 m$^2$ size with multiple households located at a distance not closer than 40 m from one another such that there is no competitive attraction induced by vision [46]. A constant number of 700 mosquitoes and around 20 individuals are used for each experimental run. To average the stochasticity due to spatial arrangement, households are randomly positioned at each successive repetition of the algorithm. Within a single run, all the households are of the same size. However, the household size varies between the runs. Seven repetitions are conducted for each of the runs to reduce the noise in the outputs. Figure 3 presents the randomly generated experimental layout.

The number of infectious mosquitoes is constant for a single experiment (since it takes a period of 10 to 12 days for parasites to reach a stage whereby they are ready for transmission). In the case when an insufficient amount of blood was consumed before the exit from the household, the mosquito starts the process of host-seeking (from the outset) except that the abandoned household is not accounted for when computing the total concentration of the CO_2_. Additionally, it is assumed that after entering a new household, the count of host-seeking time $t_{max}$ is reinitialized.

### Regression analysis of community-scale simulations

A final step of using the ABM results is to generate regression functions based on the main trends revealed by the ABM simulation results. The effects of the in situ behaviour, settlement patterns and parasite ecology are explored by fitting the response surfaces to the trends given by the simulations. That is, ABM is used as a ‘computational laboratory’ to produce data for response surfaces that capture the impact of the LLIN coverage and household size. The ABM simulations are inherently stochastic, due to the event generation by randomizing. In the community level, the uncertainty from sampled parameters at hut-level are included and a sensitivity analysis is conducted with respect to the assumed parameters using a central composite design. The assumed parameters were varied reasonably based on literature values as shown in Tables 4 and 5. The sensitivity analysis shows that the behavior of the system remains more or less the same with reasonable perturbations in the assumed parameter values. For illustration, the outputs with variability from both sampled parameters at hut level and the assumed parameters are presented, for contact and mortality rates of *An. gambiae* when confronted with LLIN treated with Aphacypermethrin chemical in Fig. 4. The outputs of the ABM simulations are averaged over 7 repetitions of the experiment. These number of repetitions was found to be sufficient by an extensive preliminary simulation. (see Additional file 1 for more ABM community-level simulation results).

A regression analysis is applied with respect to the household size and the coverage with LLINs, using the synthetic data. Given that one of the independent variables is discrete by definition, a uniform design is employed, considering household sizes of 2, 4, 6, 8 and 10, and with LLIN coverage varying from 0 to 100%. The regression is conducted in two cases: when assuming no behavioural alterations and when considering alterations caused by the parasite separately for *An. gambiae* and *An. arabiensis* when confronted with each of the chemical treatments considered.

The ABM simulation data revealed a nonlinear, quadratic relationship between the mortality rate and LLIN coverage, but an insignificant dependence of mortality rates on the household size. Consequently, the mortality rates are fitted with second degree polynomial with respect to the coverage only, see Fig. 4 for an example. Comparing the impact of the chemicals, it can be seen from Fig. 5 that in case of* An. gambiae*, Carbosulphan is the most efficient, while the other treatments display similar performances. For *An. arabiensis* the highest impact is with IconMaxx, followed by Carbosulphan. Alphacypermethrin treatment induces the lowest mortality for *An. arabiensis * of all the studied chemicals. In the case of behavioural alterations the mortality rates are similar to Fig. 5, although slightly higher, which apparently results from more frequent exposure to insecticide due to a higher number of feeding attempts.

**Fig. 9:**
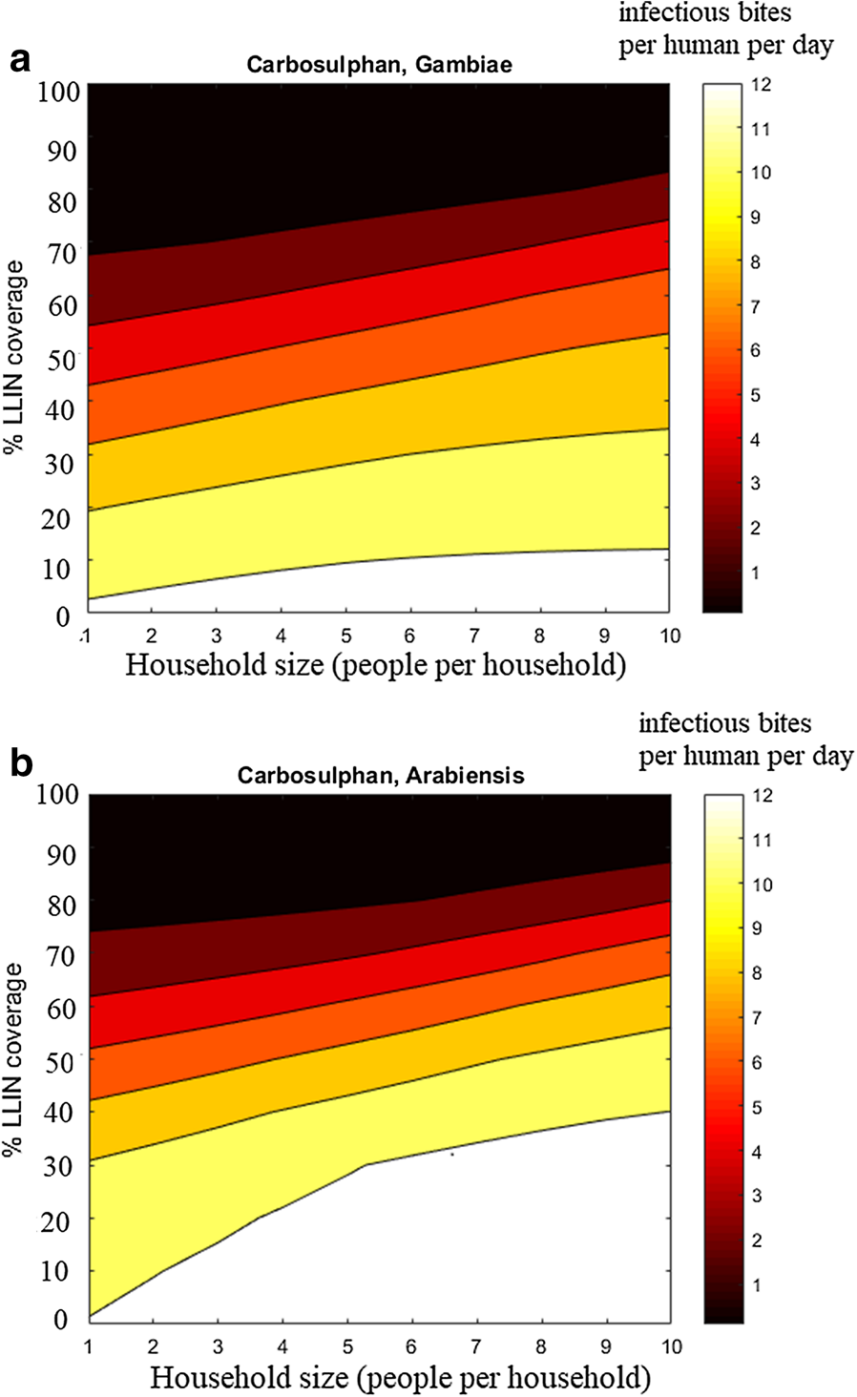
Predicted equilibrium EIR conditioned on household size and the fraction of LLIN coverage,** a** *An. gambiae* **b** *An. arabiensis* when confronted with Carbosulphan

The contact rates showed a dependency on both the household size and LLIN coverage. Moreover, for both uninfected and infected mosquitoes, the respective contact rates $\tilde{a}$ and $\bar{a}$, displayed logistic behaviour with respect to the coverage $x_2$. A certain coverage threshold was required for the contact rate to start decreasing. Hence, the logistic functions is used:

15 $$\begin{aligned} \bar{a}(x_1,x_2)&= N_b*(1 - 1./(1+\exp (-(x_2 - b_1 - b_2x_1)/b_3))) \end{aligned}$$

16 $$\begin{aligned} \tilde{a}(x_1,x_2)&= N_b*(1 - 1./(1+\exp (-(x_2 - b_1 - b_2x_1)/b_3))), \end{aligned}$$

where $x_1$ and $x_2$ denote the household size and the fraction of LLIN coverage. The values of the parameters $N_b,b_1,b_2,b_3$ are obtained from the regression fits to the ABM data, separately for each chemical. Figure 4 gives an example for one of the chemicals.

The results suggest that Alphacypermethrin displayed the highest efficiency in reducing the contact rate when applied to *An. gambiae*, while all the other chemicals demonstrate similar reduction effects. This is consistent with the confidence intervals given in [19]. On the other hand, all the chemicals feature similar performance in reducing the contact rate, in the case of *An. arabiensis*, with slightly better efficiency attributed to IconMaxx LN. Moreover, unlike the other treatments, Alphacypermethrin displays substantially better performance in the reduction of contact rates for *An. gambiae* compared to *An. arabiensis*, as can be seen from Fig. 6. The other chemicals demonstrate similar protection against both mosquito species with slightly lower contact rates when applied to *An. gambiae*.

In both cases, the contact rate tends to increase with household size for the fixed rate of LLIN coverage. Thus, the regression analysis results provide convincing evidence that lower LLIN coverage is sufficient to achieve similar reduction in contact rates for smaller household sizes, see Figs. 6, 7. For more details of the regression analysis, see Additional file 1.

## Results

### Extension to continuous time, EIR

Here, the ABM of mosquito host-seeking behaviour is linked to continuous-time compartmental modelling. This connects in situ mosquito behaviour to commonly measured quantifiers of malaria transmission, such as Entomological Inoculation Rate (EIR) and malaria incidence. A benchmark test for classical malaria models was conducted in [10], where five dynamic models of malaria transmission were tested on the basis of established performances. The most basic Ross malaria model was found to be capable of reproducing the EIR experimental data satisfactorily. Indeed, more complex models tend to suffer from poor identification of parameters and may produce results inferior to simple but more robust modelling. Following [10], the simple Ross model is considered, but utilized such that the complex factors (such as the LLIN coverage, household size or alterations of behaviour) are expressed via the ODE model parameters. That is, the regression functions from the previous section for the contact and mortality rates are substituted in place of the respective parameters in the Ross model:

17 $$\begin{aligned} di_h&= m\bar{a}b i_m(1 - i_h) - i_h r\nonumber \\ di_m&= \tilde{a} c i_h(1 - i_m) - \mu i_m , \end{aligned}$$

where $i_h$ and $i_m$ denote the fractions of infected humans and mosquitoes, correspondingly, *m* stands for mosquito-to-human ratio, *b* and *c* are the probabilities of transmission during mosquito contact with the host, $\mu$ denotes the mosquito mortality rate, and *r* stands for recovery rate for the humans. The difference to the conventional Ross model is also that the contact and death rates $\bar{a},\tilde{a}$ and $\mu$ are given by the response surfaces, fitted to various in-situ conditions. Indeed, two different contact rates, for infected $\overline{a}$ and uninfected $\tilde{a}$ mosquitoes are used in the case when alterations in mosquito behaviour is assumed. For the rest of the parameters, *m*, *b*, *c*, *r*, three sets of values were borrowed from [10], corresponding to low, medium and high transmission settings, see Table 4. The integration of the Ross model is done for household size comprising 2, 4, 6, 8 and 10 individuals while applying 20, 40, 60, 80 and 100% LLIN coverage for each household size considered.

**Table 3:** Summary of the basic agent-based model parameters, [20]

Parameter symbols	Parameter
$p_{net}$	Probability of being blocked by the physical barrier created by the net
$p_{hut}$	Probability of exiting the hut
$d_{50}$	Range of repellent coverage
$\mu _p$	Insecticide-induced death rate
*r*	Intensity of repulsion
$t_{max}$	Maximum host-seeking time (when confronted with the LLIN)
$\mu _e^G$	Rate of increase of excito-repellency for *An. gambiae*
$\mu _e^A$	Rate of increase of excito-repellency for *An. arabiensis*
$\alpha _G$	Detoxification rate for *An. gambiae*
$\alpha _A$	Detoxification rate for *An. arabiensis*
$t_{max}^{host}$	Maximal time (in minutes) spent
	Attempting to feed on protected host

The quantities of interest are the equilibrium fractions of infected mosquitoes and humans. Note that the units for mortality and contact rates are the same in both the ABM and Ross model, given as a fraction of mosquito population subject to mortality (feeding) per day. The contact rate is understood here as the average number of bites taken by the mosquito diurnally.

**Table 4:** Sensitivity design table

Range	$d_{50}$	$s_h$	$t_{host}^{max}$	$\sigma _a$
Minimum	2.7058	1.0718	0.8756	40/3
Maximum	18.2942	14.9282	25.1244	80/3

The mosquito-to-human ratio *m* is taken as a ratio of the number of humans to mosquitoes $N_m/N_h$, as given in [10]. Each value of *m* is combined with the three sets of the other parameters in Table 6, so nine pairs of equilibrium values of fractions of infectious humans $i_h^*$ and infectious mosquitoes $i_m^*$ were calculated. For each case, the response surfaces with respect to household size and LLIN protection can be now calculated.

**Table 5:** Sensitivity design table for the behavioral alteration

Range	Uninfected	Infected
Minimum	1.8934	5.8579
Maximum	23.1066	34.1421

**Table 6:** Summary of parameter selections and mosquito densities *m* from [10] used for integration of the Ross model

Parameters			
b, c, r	0.2, 0.5, 0.01	0.03, 0.275, 0.0035	0.4, 0.4, 0.05
Quantity	High transmission	Medium transmission	Low transmission
*m*	7.6	5.5	4.0

The most direct approach for estimating the overall malaria transmission in a population is by computing the Entomological Inoculation Rate (EIR) [15]. EIR is commonly measured to quantify the intensity of an infected mosquito pool and its propensity to transmit malaria infection to human populace within a given time period. Conventionally, the EIR is measured per period of time: per night, monthly, seasonally or annually. The transmission patterns represented by the pair of EIR and Parasite Rate (PR) depend on a number of ecological, climatic and socioeconomic factors [49]. Here, the simulation results are compared to the experimental results reported in [50], where a trend curve together with the 95% confidence interval was created using data from 31 sites in Africa. At the time when the survey was published, the annual EIR varied from less than 1 to more than 1000 infective bites per person per year. The transmission patterns represented by the pair of EIR and Parasite Rate (PR) depend on a number of complex factors, such as ecology, climate and socio-economic development [49]. By integration of the modified Ross model, the impact of partial population coverage with LLIN, alterations in mosquito behaviour and household size, on EIR and PR, can be quantified. These two factors are computed from equilibrium fractions of infectious mosquitoes $i_m^*$ and humans $i_h^*$, i.e., by the steady state of the Ross model. As EIR is defined as the product $mai_m^*$, a direct computation gives:

$$\begin{aligned} EIR= m \bar{a} i_m^* = \frac{\bar{a}\tilde{a} bcm - \mu r}{\mu b + \tilde{a} b c}, \end{aligned}$$

where

$$\begin{aligned} i_m^* = \frac{\bar{a}\tilde{a} bcm - \mu r}{\mu m \bar{a}b + \bar{a} \tilde{a} b c m}. \end{aligned}$$

As a result, three pairs of equilibrium EIR and malaria incidence $i_h^*$ correspond to each of the original selections of parameters given in [10], see Fig. 8. In addition, the EIR and PR values for those LLIN and household values for which the regression models of $\bar{a},\tilde{a}, \mu$ were calibrated, can now be computed. These values, as continuous functions of LLIN, are added in Fig. 8. Figure 9 gives an example of the response surface of the EIR values as a function of household size and LLIN coverage. Respective figures for all the chemicals are given in Additional file 1.

Note that all the results presented in this paper are based on data from [19]. Additional experimental data would improve the reliability of the results, especially for the behaviour of mosquitoes between the households. Given that similar data are available elsewhere, the approach allows general trends and response surfaces to be produced based on such data in an analogous way.

## Discussion

Transmission of diseases depends on complex factors, medical, environmental or socio-economic, to mention a few. A serious issue of simulating such processes by traditional population-level compartmental models is the calibration; the models tend to get very complex, overloaded with unidentifiable parameters. The situation is typically made worse by the scarcity of real data needed for the calibration.

An approach to combine complex *in situ factors together with classical compartmental models, in the case of Malaria transmission, is presented*. The idea is to simulate the individual level processes by discrete ABM calculations under varying conditions for the factors considered. The resulting data is used as input for regression to quantify the impact of the factors as response functions. The key coefficients of a compartmental model can then be expressed by these functions.

Naturally, the underlying ABM model needs to be carefully calibrated. This is only possible if sufficient in situ data is available, and the ABM model is parsimonious enough. Such a model is developed and is calibrated using extensive MCMC (Markov chain Monte Carlo) simulations against a set of field data. The simulations can be extended to community level to study the impact of intervention levels and basic socio-economic factors. It appears that even if all the ABM model parameters are not well identified, the randomized simulations provide consistent trends with respect to the factors studied: the LLIN coverage, various chemicals, household size and behavioural changes of infected mosquitoes.

While the present work should be interpreted as a proof of concept, based on one set of field data only, certain interesting conclusions can already be drawn. A lower LLIN coverage is sufficient for smaller household sizes in order to attain a certain reduction in the biting rate. The contact rates are higher when assuming behavioural alteration, but with high LLIN coverages the contact rates become virtually the same, i.e., the effect of alterations in mosquito behaviour due to the presence of the *Plasmodium* parasite becomes negligible. The difference between mosquito species is evident as well. The coverage required to achieve similar reduction in the number of infectious contacts is higher for *An. arabiensis* than *An. gambiae*, basically due to the lower death rate of *An. arabiensis*. The death rates of both species increase when considering the alterations in behaviour. An intuitive explanation is the more intensive exposure to insecticide for infectious mosquitoes, due to increased attempts to feed on multiple hosts during the night.

Different values of the Ross model parameters can result in the same EIR values, which prevents the identification of transmission factors based on EIR data alone. The agent-based model gives an approach which incorporates the in situ data with contact and mortality rates. So the overall transmission characteristics can be estimated by including various features that impact the EIR and malaria incidence, e.g., by reducing the mosquito–human contact rates and increasing the mortality through control measures or socio-economic factors. Additionally, local characteristics can be combined with spatially explicit model that accounts for heterogeneity in human and mosquito distribution, see [23].

The present study can naturally be extended in several ways. In addition to *An. gambiae* and *An. arabiensis*, other mosquito species can be be considered, as well as intervention methods other then LLINs. Here, constant values are assumed for the mosquito density *m*, although it actually is seasonally varying due to rainfall and temperature. Spatial aspects like the local disposition of mosquito-breeding sites can be included by calibrating the respective parameters to be site dependent. This way, the modelling can be extended to larger geographical areas. The mosquito–human contacts with an infected mosquito are assumed here to be equally infectious, whereas some people might have acquired partial immunity either by constant exposure to the parasite or by artificial means via vaccines [51]. Thus, the impact of naturally acquired immunity can be incorporated in the model by treating the hosts as a population of agents as well, and making the transmission parameter *b* dependent on the individual immunity level. Also, the present study is restricted to night-time in-house biting scenarios. The model can be improved to include outdoor biting scenarios [52]. All such extensions are technically feasible but require sufficient field data for a robust calibration of an underlying ABM model. Under this condition, agent-based models are capable of generalizing various effects from the in situ level to continuous modelling.

## Conclusions

The common pitfall of obtaining data that could be directly used for model calibration in malaria transmission modelling, may be overcome by linking in situ field data with continuous malaria models. Thus, complex phenomena such as the impact of the coverage of the population with long-lasting insecticidal nets (LLINs), changes in behaviour of the infected vector and the impact of socio-economic factors can be included in continuous level modelling. The computational approach is generic, and can be applied to other cases where suitable in situ data is available.

## Supplementary Information

**Additional file 1.** From in situ to continous model. This file contains more detailed explanation of the data and likelihood, model calibrations, Regression with simulated ABM outputs, the extension to continuous times and a detailed description of the ABM using the ODD protocol.

## Data Availability

The datasets used and/or analysed during the current study are available from the corresponding author on reasonable request.
