# Supplementary material for: Agent-based modelling of complex factors impacting malaria prevalence
Source: Malar J. 2021 Apr 15;20:185. doi: 10.1186/s12936-021-03721-2 (PMC8048062; doi:10.1186/s12936-021-03721-2)
Supplement: Supplementary file 1 — Additional file 1. From in situ to continous model. This file contains more detailed explanation of the data and likelihood, model calibrations, Regression with simulated ABM outputs, the extension to continuous times and a detailed description of the ABM using the ODD protocol. [file 12936_2021_3721_MOESM1_ESM.pdf]

Additional file 1

Miracle Amadi Anna Shcherbacheva Heikki Haario

A Data and Likelihood function

The hut experiment data from [1] contains the percentages of exited, blood-fed and dead mosquitoes for each chemical tested. The results were collected for the mosquito species: *An. gambiae* and *An. arabiensis*. The outcomes in each case were averaged over six repetitions of the experiment, in order to reduce the uncertainty attributed to the differences in an individual host's attractiveness. The data and the 95% confidence intervals are shown below

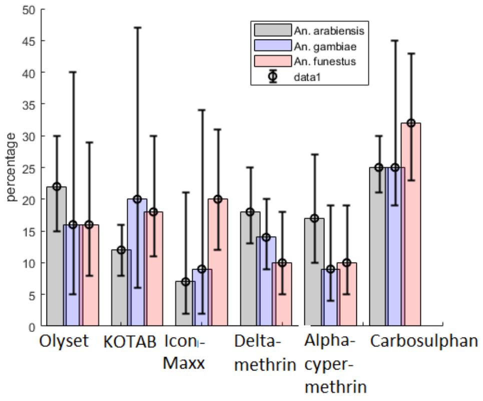

(a)

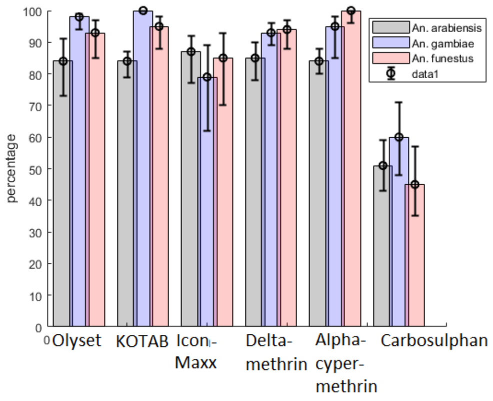

(b)

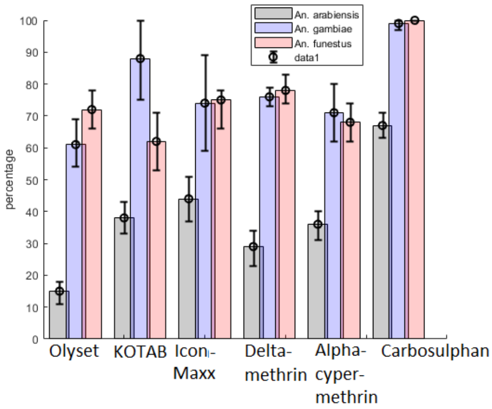

(c)

Figure S1: Summary of data from [1] for several insecticidal treatments. Percentages of (a) blood-feed, (b) exited (c) insecticide-induced mortality rates of different mosquitoes. The black bars indicate 95% confidence intervals.

The simulated and the experimental data are compared using the sum of squares cost function, see Equation S1. The likelihood is assumed to be Gaussian, due to repeated measurements. Uninformative uniform priors are chosen for sampling in all the cases considered, since no prior estimate of the parameters is available. The likelihood is sampled using adaptive MCMC from [2] to approximate the posterior distribution of the model parameters, i.e., to determine how well the available data can identify them. The sampled parameter sets represent the model evaluations that produce values within the noise level of the data. The sum of squared difference between the observations and the model outputs (the negative log-likelihood) is given by

$$\mathbb{S}_{sum} = \sum_{i=1}^{N_r} \frac{(Y_i - \hat{Y}_i)^2}{\sigma_i^2}. \tag{S1}$$

The standard deviations are chosen so as to agree with the confidence intervals shown in Fig. S1. Here, $N_r = 6$ gives the number of measured responses, the exit, fed and mortality rate each for the two species of mosquitoes considered. It should be noted that the data from [1] (see Fig. S1)is relative so the size of the mosquito swarm used in the simulations can be chosen by numerical efficiency. Since the model is stochastic, all the results are averaged over multiple repetitions. The combination of 6 repetitions and a swarm of 600 mosquitoes turned out to result in a reasonably small variance at a minimal CPU time. The total wall-clock time for one evaluation of the cost function, using parallel GPU calculations, was approximately 2 seconds as performed

on a CPU core-i7 2500K, GPU GetForce GTX TITAN.

B Model calibration

The model parameters are calibrated against data on behavioural differences in host-seeking of *An. gambiae* and *An. arabiensis* upon confrontation with ITN/LLIN, using extensive MCMC (Markov chain Monte Carlo) simulations.

In a preliminary step, the distribution of two parameters, the probability of penetration through the net, $1-p_{net}$, and probability of exiting from the hut, p_{hut}, were sampled by data from control tests using nets without chemicals.

All the chemical treatments considered have a short spatial range of action (approximately 10cm), which is modelled by the value of the parameter s representing the spatial spread of the chemical. This value is kept fixed in the simulations. The rest of the parameters; those defining the net repellent, insecticide-induced death rate and insecticide-induced exit rate, $r, \alpha_G, \alpha_A, \mu_e^G, \mu_e^A$, are sampled by MCMC. Most of the parameters are identified rather well. Some of the parameters were not accurately identified in preliminary samples. However, despite the uncertainties in the parameter distributions, the overall behaviour of the model still remains statistically the same. It appears that these parameters do not impact much on the model, and they could hence be fixed. The calibration results is presented for only one chemical Alphacypermethrin, other results are available upon request.

The model calibration results overall show a good fit to the response measurements, and the variability of the simulated model outputs match with the error bars of the measurement. The fit is evident in Fig. S2, where the blue lines denote the error bounds of the actual measurement, depicted by the black constant line. The pairwise correlation plots (see Fig. S3) mostly do not reveal any consistent correlation between the sampled parameters. On the other hand, for Alphacypermethrin, the chemical repulsion parameter is strongly inversely proportional to the detoxification rate for *An. gambiae*, while no similar relation is revealed in the case of *An. arabiensis*. This may be because Alphacypermethrin features a low blood-feeding rate with high exit for *An. gambiae*, so that, given a fixed period before the exit (by fixing parameter μ_G), the chemical is either highly repulsive and moderately detoxified (high r and low α_G), or alternatively, subject to intensive detoxification but lower repulsion (low r and high α_G), which results in a similar mortality

rate, since the total accumulated dosage is similar in these two cases. The mortality rate for *An. gambiae* with Alphacypermethrin produced with the sampled values of parameters displays a high variation from the sampled mean of the model output, which in some cases is even comparable with that of *An. arabiensis*. However, the mean values of the model outputs are all within the confidence bounds.

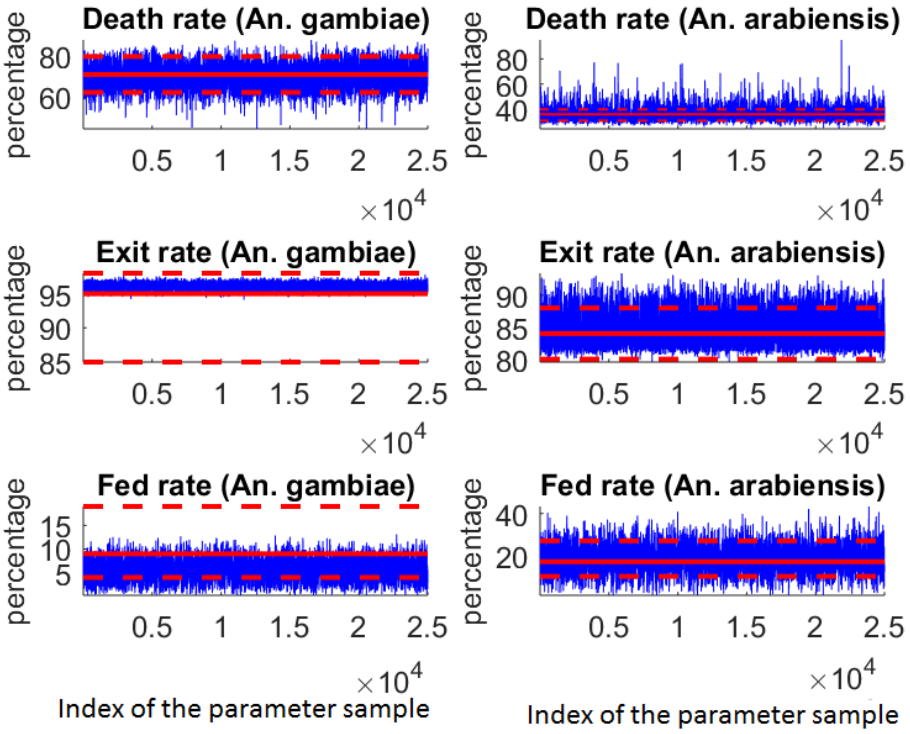

Figure S2: Results of MCMC model calibration with experimental data reported in [1], where LLIN is impregnated with Alphacypermethrin. Model outputs obtained using a posterior distributions of parameters (blue trace lines) versus the data (mean values in red solid and 95 % confidence intervals in red dashed lines).

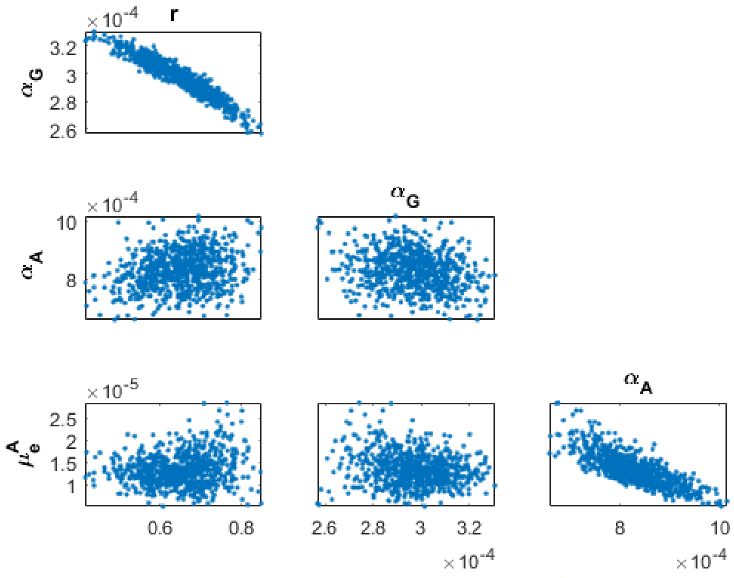

(a)

Figure S3: Results of MCMC model calibration with experimental data reported in [1], where the LLIN is impregnated with Alphacypermethrin. (a) Parameter chains and (b) Pairwise marginal posterior distributions of model parameters.

C Regression Analysis, contact and mortality rates

The ABM model was calibrated for the situation with one person in hut. However, the simulations can be performed for several persons in one household, or a given number of people divided in households of different sizes. A few additional assumptions, based on literature, further allow to approximate the impact of complex phenomena such as how the parasite changes the behaviour of the vector. The simulations are performed under different fractions of the hosts covered by the LLINS, using different chemicals. Repeated randomized simulation experiments provide data from which the overall impact of such factors can be extracted by regression analysis. Especially, the interest is in the biting rates and mosquito mortality, as they appear to be the key coefficients in continuous-level compartmental models.

Fig. S4 shows simulated contact rates for both uninfected $\tilde{a}$ and infected $\bar{a}$ mosquitoes, when the LLIN coverage ranges from 0 to 100 %, for household sizes from 2 to 10 persons. Logistic functions are fitted to the data.

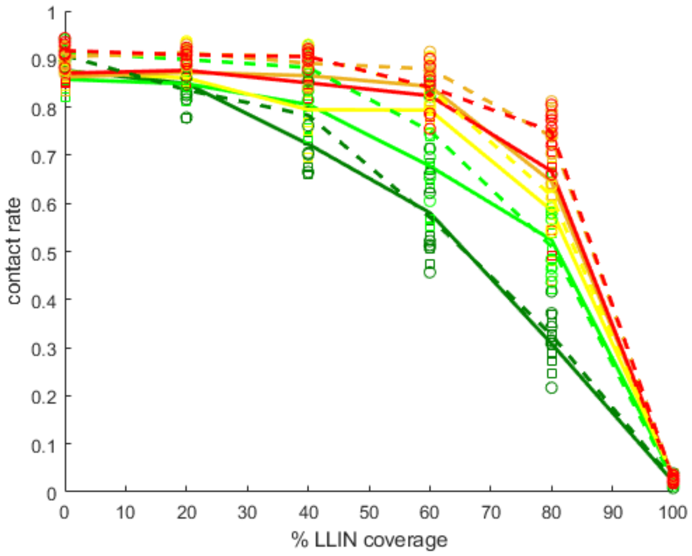

Figure S4: Simulation data from ABM for the contact rates for uninfected (solid lines) and infected, with behavioural alterations, (dashed lines) mosquitoes for different household sizes: 2 (dark green) , 4 (green), 6 (yellow), 8 (dark yellow) and 10 (red) people, for *An. gambiae* and LLIN treated with Carbosulphan. Diamonds and dots denote repeated simulation results for each pair of household size and coverage averaged (solid and dashed lines) over 7 repetitions for infected and uninfected mosquitoes

Unlike the contact rates, the mortality rates are estimated to be dependent only on the LLIN coverage. The regression is conducted in both cases: when assuming no behavioural alterations and when considering alterations caused by the parasite, separately for *An. gambiae* and *An. arabiensis* when confronted with each of the chemical treatments considered. Mortality rates are fitted with a second degree polynomial with respect to the LLIN coverage.

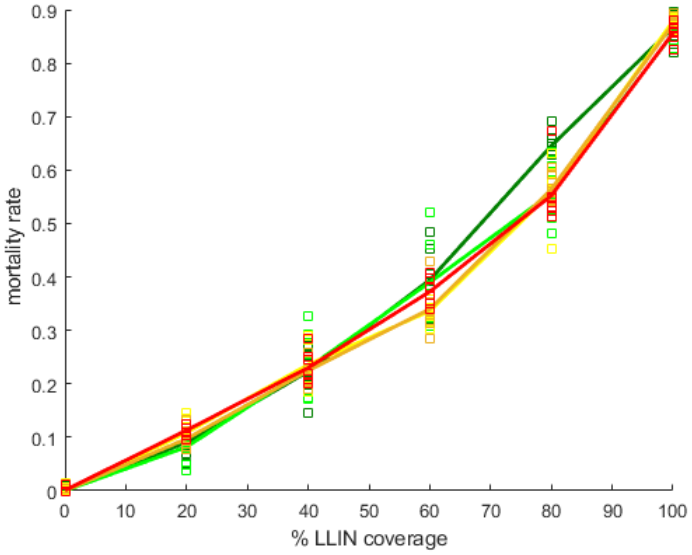

Figure S5: Simulation data from ABM for the mortality rates for *An. gambiae* and LLIN treated with Carbosulphan, conditioned on the coverage when assuming no behavioural alterations by parasite: 7 repetitions for each pair of household size and coverage (dots) and the rates averaged over the repetitions (solid lines).

D Extension for continuous time

Here the dependence of EIR on the LLIN coverage and household size is presented. Fig. S6 suggests that the coverage required to achieve similar reduction in the number of infectious contacts is higher for *An. arabiensis* than *An. gambiae* for all of the chemicals, except IconMaxx. The difference is more noticeable for high household sizes. The surfaces are plotted for the case of behavioural alterations in mosquitoes. However, they are qualitatively similar to the case of no alterations, but the absolute values of EIR are lower in the latter, as can be seen from the predictive distribution (see Fig. 8 of the main text).

Further, the equilibrium EIR and malaria prevalence are compared for both *An. gambiae* and *An. arabiensis* (see Fig. S7). It can be seen from the plots in Fig. S7 that the level of coverage required for total elimination of the disease is consistently higher in the case of behavioural alterations induced

by the parasite. Additionally, the values for both the EIR and i_h^* are lower for *An. gambiae* than *An. arabiensis* for the same rate of coverage. This can be attributed to the fact that *An. gambiae* is more susceptible to chemical influence than *An. arabiensis.*

Comparing the mortality rates with the equilibrium fractions of infected mosquitoes i_v^*, it can be seen from Fig. 5 of the main text and Fig. S7 (e and f), that higher chemical toxicity entails lower equilibrium proportion of infected mosquitoes. Carbonsulphan for instance, being the most toxic chemical for *An. gambiae*, correspondingly features a lower equilibrium proportion of infected mosquitoes. The same scenario is virtually applicable to all other chemicals and for *An. arabiensis.* Again, as can be seen from Fig. S7, EIR displays a substantial decrease with increase in LLIN coverage, which occurs due to increasing mortality, whereas i_h^* starts to decrease only after a certain threshold coverage. This latter tendency can be traced to the mechanism inherent in Equation 17 (from the main text), which suggests that a drastic reduction in malaria prevalence occurs only when the number of infectious bites per human per time unit (which essentially comprises the EIR) decreases below the threshold of 1 [3].

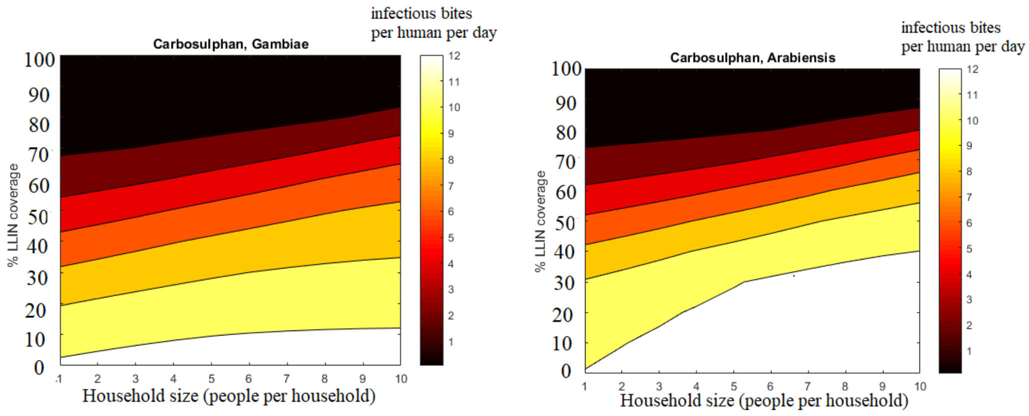

(a) (b)

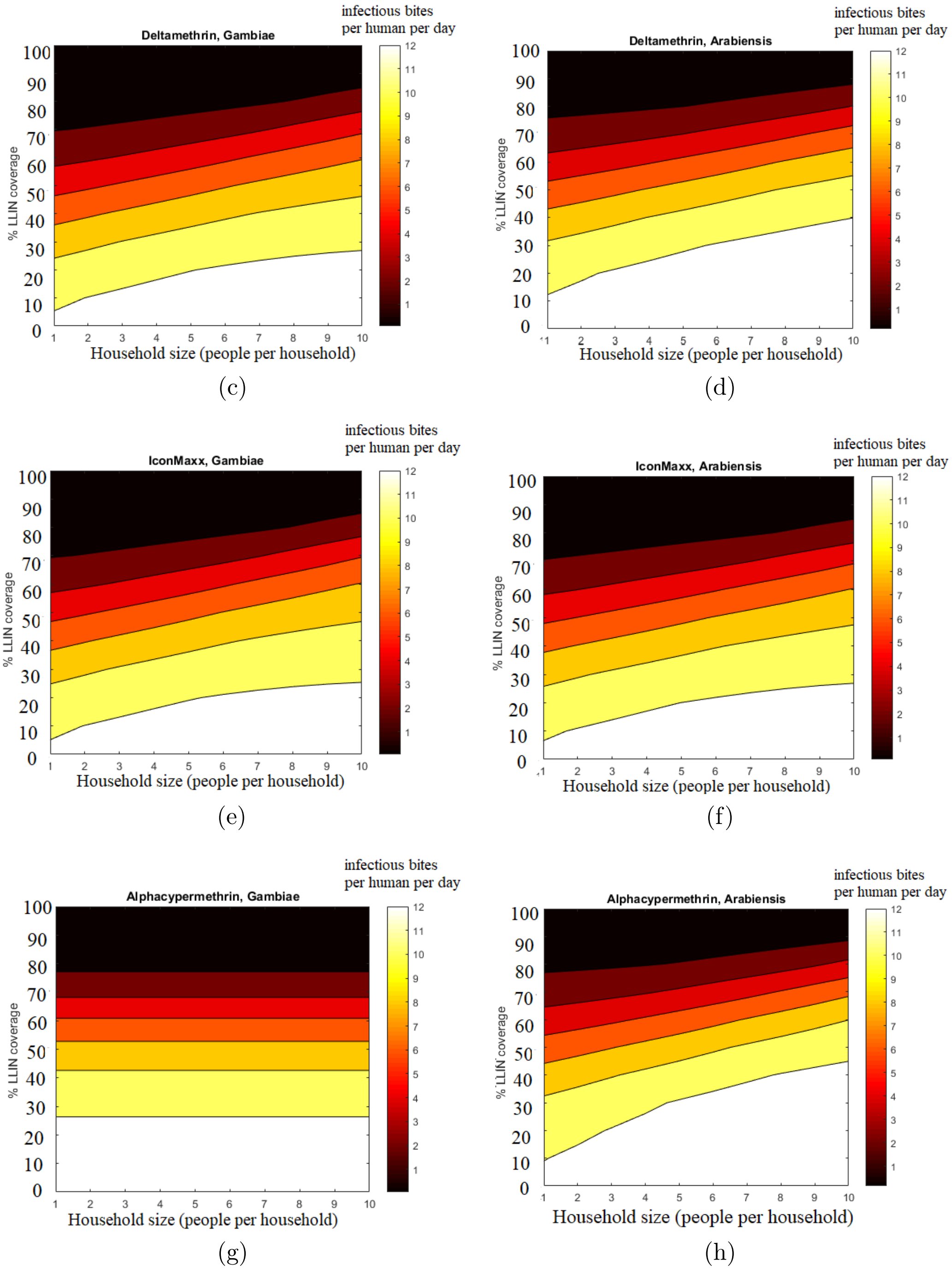
Deltamethrin, Gambiae
Deltamethrin, Arabiensis
IconMaxx, Gambiae
IconMaxx, Arabiensis
Alphacypermethrin, Gambiae
Alphacypermethrin, Arabiensis
infectious bites
per human per day
% LLIN coverage
Household size (people per household)

(c) (d) (e) (f) (g) (h)

Figure S6: Predicted equilibrium EIR conditioned on household size and the fraction of LLIN coverage for *An. gambiae* and *An. arabiensis* when confronted with each of the chemicals considered in the study. The response surfaces are given for the case of behavioural alterations for the parameter set and mosquito density corresponding to high transmission setting, for the following combinations of chemicals and mosquito species: (a) Carbosulphan and *An. gambiae*, (b) Carbosulphan and *An. arabiensis*, (c) Deltamethrin and *An. gambiae*, (d) Deltamethrin and *An. arabiensis*,(e) IconMaxx and *An. gambiae*, (f) IconMaxx and *An. arabiensis*, (g) Alphacypermethrin and *An. gambiae*, (h) Alphacypermethrin and *An. arabiensis*

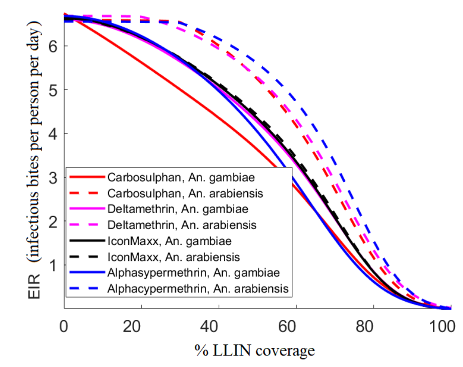

(a)

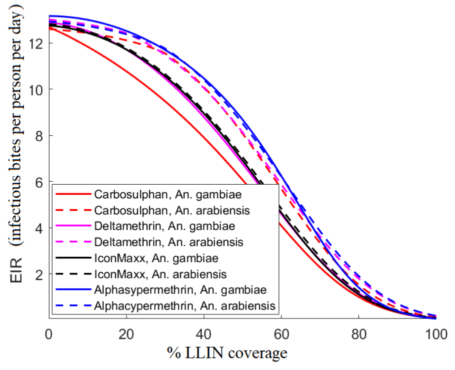

(b)

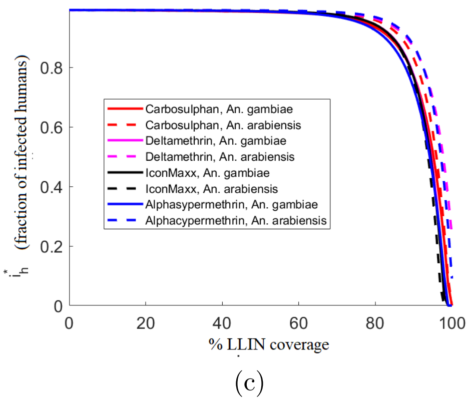

(c)

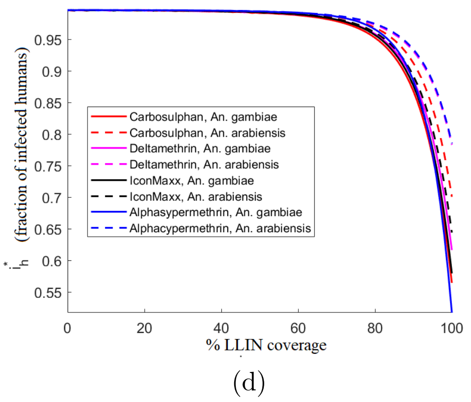

(d)

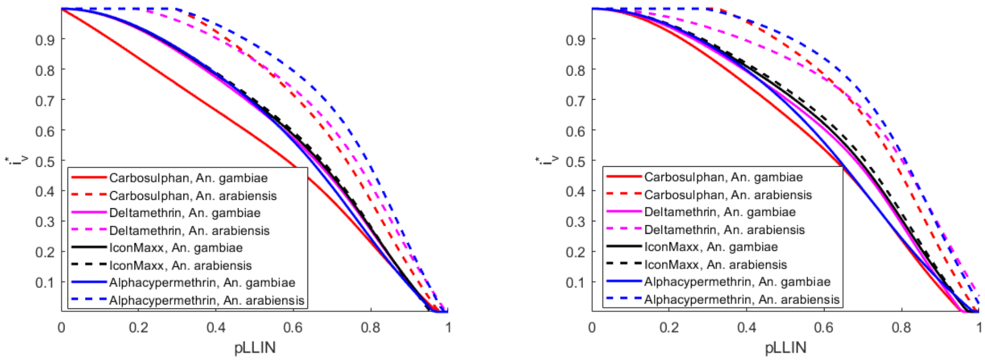

(e) (f)

Figure S7: Equilibrium values of Entomological Inoculation Rate (EIR), malaria prevalence i_h^* (fraction of infected humans) and fraction of infected mosquitoes conditioned on the partial coverage of LLIN (pLLIN) for *An. gambiae* and *An. arabiensis* when confronted with the chemicals under study for the parameter set and mosquito density corresponding to high transmission setting: (a) EIR in the case of no behavioural alterations; (b) EIR in the case of behavioural alterations; (c) i_h^* in the case of no behavioural alterations; and (d) i_h^* in the case of behavioural alterations (e) i_m^* in the case of no behavioural alterations; and (f) i_m^* in the case of behavioural alterations.

E ODD PROTOCOL

Here, the discussion of how the ABM simulations were done and how the observed results emerge, together with their impact on the regression analysis done in Sections C and D, is presented.

E.1 Purpose and patterns

E.1.1 Purpose

The purpose of the model is to provide data reflecting the impact of various complex factors affecting malaria such as household size, LLIN coverage, and alterations in mosquito behaviour induced by malaria parasite, in the form that can be directly used by the continuous models of malaria. Thus, the

ABM simulations is used as a 'computational laboratory' where data can be produced for regression analysis, so as to enable the calibration of the key parameters of classical malaria models. In considering these factors, the modelling of a single host in the hut is done, followed by the household level modelling, with multiple individuals sleeping under the same roof. Subsequently, the household-level model is extended to community-level scenarios, enabling simulations of heterogeneity of mosquito-to-human contact rates due to partial coverage with nets or different household sizes.

E.1.2 Pattern

The hut-level simulations are data driven as they aim to reproduce the patterns of the data employed from [1]. In the community-level case, since there is no data to calibrate the simulations, the literature values are employed and a sensitivity analysis is conducted based on these values to ascertain how the assumed parameter values impact the overall outcomes. See Table S2.

E.2 Entities, state variables, and scales

E.2.1 Entities

The entities in the model include humans, mosquitoes, nets and chemicals. Humans are modelled as individual agents, attributed with the state of infection, the use of insecticidal nets and spatial position. Humans do not carry out any actions and their features are constant in time. This is because the main focus of the model is to control the mosquito population. Two female mosquito species were employed: *An. gambiae* and *An. Arabiensis.* Given that only *female* mosquitoes transmit the parasite during blood-feeding, and mating is outside the scope of this project, the ABM simulations do not include the *male* mosquitoes. The difference of the species is attributed by their host-seeking behaviours (anthropophilic or opportunistic preferences of mosquitoes) when confronted with the insecticidal nets. The mosquitoes can either be infected or uninfected. The infected mosquitoes differ from the uninfected ones in terms of their biting habit. The number of infectious mosquitoes is constant for a single experiment. This is because, it takes a period of 10 to 12 days for parasites to reach a stage whereby they are ready for transmission whereas the ABM simulation in this study is only for a night.

Insecticidal nets of 1.5m width, treated with four different chemicals: Carbonsulfan, Iconmax, Alphacypermethrin and Deltamethrin, are simulated. The difference between these chemicals is represented by their impact (in terms of contact irritancy, excito-repellency and poisoning) on each of the mosquito species under study. Considering that some of the bed nets used in rural communities are typically holed in a practical way, purposely holed nets are widely used in hut trials [4]. Therefore, broken nets are simulated in such a way that the likelihood of mosquito penetration is non-zero. For the hut-level case, the human agent is always covered with the net. But at household and community levels, the number of protected humans can vary from 20 to 100% and remains constant throughout the simulation. Additionally, a hut barrier (walls) is simulated for each of the huts. In the hut-level experiment, the walls have window traps from-which mosquitoes can exit (see [1]). In the community-level experiment, a usual human dwelling is modelled.

E.2.2 State variables

For each of the mosquito agents, properties are individually assigned and updated within the simulation (see Table S1).

Table S1: Property list of each agent and the relevant model component.

Property	Model component	Type
Spatial position	Motion	Set of coordinates
Inside/Outside the hut	Motion	Binary
Inside/Outside the net	Motion	Binary
Trapped	Motion	Binary
CO_2 concentration	Motion	Float
Fed	Host-seeking	Binary
Time indoors	Host-seeking	Integer
Klinotaxis	Host-seeking	Binary
Dead	Death (Poisoning)	Binary
Accumulated dosage of chemical	Poisoning	Float

E.2.3 Scale

Hut scale The mosquitoes are initially represented in the simulations as a number of agents in a rectangular patch of 3m (which is a typical experi-

mental hut-size [5]) at uniformly random spatial positions.

Community scale In the community-level simulations, mosquitoes are randomly positioned inside the simulated transmission domain of 25600 m^2 size with multiple households located at a distance not less than 40 m from each other so that there is no competitive attraction caused by vision [6]. The hut-size for the household-case is 13 m.

Time scale The ABM simulations cover a period of one night (10 hours) plus a 24-hour additional delayed mortality. Each calculation simulates an experiment of 34 hours.

E.3 Process overview and scheduling

E.3.1 Processes

The model describes and calibrates mosquito's responses and behaviours based on four basic components: motion, host-seeking, poisoning and death (see Table S2), where each of the components has a number of related features. The movement and host-seeking behavior of the mosquito is governed by an attraction model, based on the assumption that a mosquito estimates the direction of odour increase (the gradient) from the host via the klinotaxis mechanism [7]. As the mosquito approaches the host, the likelihood of accepting steps away from the host reduces (see Fig. S10b). The current spatial position of the mosquito is updated at every time step.

Apart from the physical material barrier posed by the insecticidal nets, they are also equipped with poisoning and repellent effects. A mosquito is said to be exposed to the poison upon contact with the net surface (see [8]). Again, the explanation of detoxification is considered, that the chemical concentration accumulated in mosquito body is exponentially decaying with a rate which is dependent on both the chemical and mosquito species [9], [10]. The total accumulated dosage of poison which depends on the number of contacts with the net and the detoxification rate, is updated for every mosquito at each time step, and determines their probability of death. Additionally, the delayed mortality that is as a result of the prolonged impact of poison in mosquitoes, is accounted for. If mosquito is marked as dead, the mosquito is

removed from the simulation such that no properties of the mosquitoes are updated again. The repulsion effect amplifies as the mosquito approaches the source of repellent (see Fig. S11). This repulsion effect influences the mosquitoes decision of approaching the host and can induce early exit from the hut. If a mosquito exits, no other property of such mosquito is updated in the simulation except for their mortality status which is updated after 10 hours, and tracked for 24 hours. This is because the delayed lethal action of the chemical is assumed to start after the 10 hours in the hut.

A mosquito is scored as fed if it penetrates through the net and its updated position is very close to the host (a minimal distance ϵ between a mosquito and the host is defined). In the hut-level case, a mosquito can take only one bite since there is only one human. But in the household and community-level case, the mosquito can take several bites; in this case, up to 5 bites. In household and community-level simulations, the tendency of mosquitoes to switch to neighboring individuals after spending a certain period of time in unsuccessful attempts to feed on a protected human, is also considered. Hence, in any case, The mosquito switches to a pure random walk, without any control of attractive odor, if the maximum number of bites is reached or if the maximum time a mosquito can spend on host-seeking is used up. Nevertheless, the barriers raised by the net and the repellent effect alongside the effect of chemical poisoning remains functional under this condition. For all mosquitoes that are inside the hut and are not dead or exited, the time spent indoors are updated at each time step. In community-level case, if mosquito consumes an insufficient amount of blood before exiting the household, the mosquito begins the host-seeking process from the outset, except that the abandoned household is not accounted for when the total concentration of the CO_2 is computed. Additionally, it is also assumed that the host-seeking time count is reinitialized after entering a new household. See Fig. S8 for a diagrammatic explanation of the above discussed processes.

E.3.2 Schedule

The update of the property list of mosquitoes happen at the same time after each time step. One iteration step in the simulation corresponds to 2 seconds. The basic algorithm for the execution is given in item E.1.

Table S2: Modelled processes

Model component	Attributes	Definition
Host-seeking		
	· CO_2 concentration, Klinotaxis	Equation S3
	· Distance-dependent attraction	Equation S5
	· Host seeking time	
Motion		
	· Random walk, accept/reject steps	Equation S4
	· Excito-repellency	Equation S11
Poisoning		
	· Accumulation of the chemical dosage	Equation S7
	· Detoxification	Equation S8
Death		
	· Natural mortality	Equation S6
	· Insecticide-induced mortality	Equation S9
	· Delayed mortality	Equation S6 with model extension

Algorithm E.1 Model algorithm

1. Propose candidate position $\mathbf{x}^n$ by adding a stochastic increment to the previous position, i.e., compute $\mathbf{x}^n$ by Equation S2;
2. Account for natural mortality. Generate random number $u \sim U[0,1]$. Remove the agent if $u < \alpha^{\Delta t}$;
3. Account for insecticide-induced mortality. Generate random number $u \sim U[0,1]$. Remove the agent if $u < \alpha_p^{\Delta t}$;
4. Evaluate the CO_2 concentration $C(\mathbf{x}^n)$ at new position $\mathbf{x}^n$ as given in Equation S3;
5. Compute the scaling factor $\sigma_{acc}(\mathbf{x}^n)$ as given by Equation S5;
6. Recalculate the scaling factor, while considering the *excito-repellency*, conditioned on the amount of accumulated chemical by Equation S11;
7. Compute probability of acceptance by attraction, $\alpha_a(\mathbf{x}^n|\mathbf{x}^{n-1})$ for position $\mathbf{x}^n$ by Equation S4;
8. Compute the probability of rejection α_{rej} resulting from repellent $\alpha_r(\mathbf{x}^n|d_p, s)$ by Equation S10;
9. Generate random number $u \sim U[0,1]$, if $u < min\{1, \alpha_a(1-\alpha_r)\}$, mark position $\mathbf{x}^n$ as preliminarily accepted, otherwise, mark position as rejected and remain at the old position $\mathbf{x}^n = \mathbf{x}^{n-1}$;
10. Account for the physical net barrier. If candidate step $\mathbf{x}^n$ is inside and old position $\mathbf{x}^{n-1}$ is outside of the net, and position $\mathbf{x}^n$ was preliminarily accepted, generate random number u. If $u < 1 - p_{net}$, accept the new position $\mathbf{x}^n$. Otherwise, select the closest point on the net $\mathbf{x}^{net}$ to $\mathbf{x}^{n-1}$ and assign new position $\mathbf{x}^n = \mathbf{x}^{net}$;
11. Account for the wall barrier. If candidate step $\mathbf{x}^n$ is outside and old position $\mathbf{x}^{n-1}$ is inside of the hut and position $\mathbf{x}^n$ was preliminarily accepted, generate random number u. If $u < p_{hut}$, accept the new position $\mathbf{x}^n$. Otherwise, chose closest point on the wall $\mathbf{x}^{wall}$ to $\mathbf{x}^{n-1}$ and assign new position $\mathbf{x}^n = \mathbf{x}^{wall}$;
12. Update the total accumulated chemical dosage C_{tot} by Equation S8;
13. Account for *detoxification* of the total accumulated chemical dosage C_{tot} with the rate α;
14. Update the property list of mosquito;
15. Move to step 1, $n \to n+1$

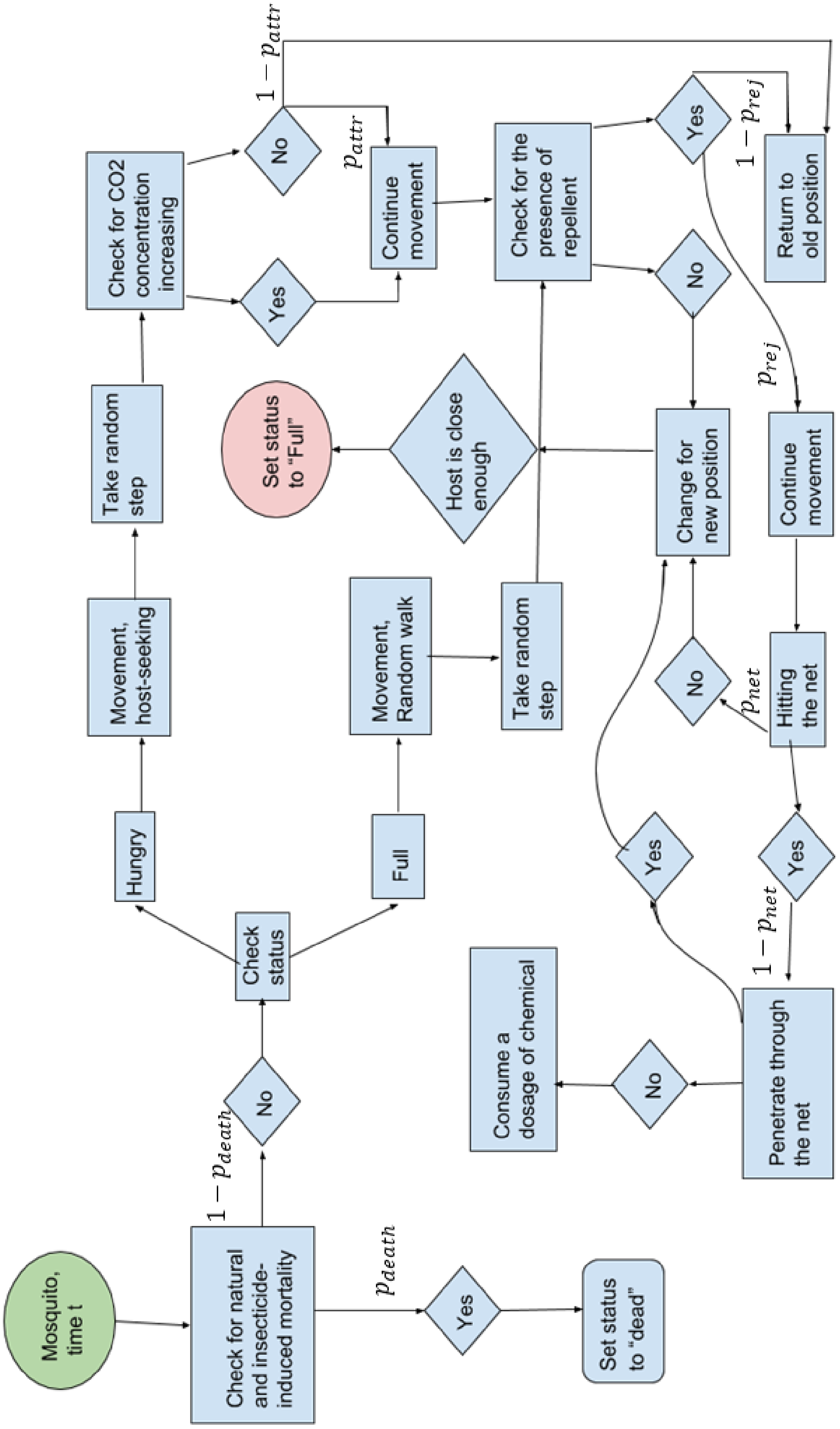

Figure S8: A decision tree showing the key features of the ABM algorithm of mosquito host-seeking actions in the presence of the LLINs introduced in [11]. Here, some of the choices are probabilistic, depending on the state of the agent. p_{death} denotes the probability of death, p_{net} stands for the probability of being blocked by the physical net barrier, p_{attr} denotes the probability of accepting the proposed step, p_{rej} stands for the probability of rejecting the proposed step due to the repellent effect.

E.4 Design concepts

E.4.1 Basic principles

Mosquito movement and attraction model The mosquito attraction model is based on the assumption that a mosquito estimates the direction of odour increase (the gradient) from the host via the klinotaxis mechanism [7]. During this plume-tracking activity, the mosquito samples the host odour at one location, changes location and then repeats the sampling, and uses its memory of the concentrations previously observed to select the next position [12],[13]. In the present work, the space is continuous, and the movement of mosquitoes is guided based on Euclidean distances to the humans and households. In the absence of the sensory signals, the movement of mosquito constitutes pure random walk, which is typical for the ABMs that include animal navigation, ([14]). Imitating this mechanism, flight of mosquitoes is modelled as a discrete-time correlated random walk. However, when there are attraction effects, sufficiently close for sensing the host, the main features of the Metropolis algorithm are employed to simulate the random walk directionally biased by attraction [15]. The Metropolis algorithm features an *accept/reject* movement. After a random candidate position is proposed by the Brownian motion, the probability of accepting the new position for a given agent is defined to favour candidate steps taken in the direction of increasing concentration of CO_2, i.e., towards the attraction source, (see [15]). In addition, the acceptance probability is also influenced by the presence of treated nets and the barrier imposed by the walls in human dwelling. These effects are incorporated by a rejection function. The concentration of attractive odour and the area covered by the odour is modelled using the diffusion equation solution, taking into account only the diffusive spread of the odour. The effect of wind is ignored for simplicity. Thus, the region of high odor concentration may be assumed to be a specific location where the host is located and the maximum distance at which the mosquito is able to detect the host, is seen as the region of low concentration. This is consistent with the principle of the diffusion equation that describes the expel of the flow of certain quantities (intensity, temperature) over space [16].Therefore, the Gaussian Kernel centered around the host's spatial location is used. Naturally, when other significant factors influencing the dispersion of mosquitoes are taken into account, the concentration may be defined in a different way, such as using advection-reaction-diffusion equations, which includes the flow of air and intermittent concentration plumes etc.,(see [13]). The concentration that allows mosquitoes to sense humans in the household and community-level

case, is calculated similarly to the case of a single person, i.e. as a Gaussian, with the argument given by a weighted sum of the individual distances from the position of the mosquito to the location of each of the hosts . The total attracting concentration is based on the principle of the function *soft-max*, which has been widely implemented in machine learning and neural networks, (see [17], [18]). The weight Wn is added to account for the fact that, depending on the mosquito species, the response of a mosquito to the cue emitted from households increases at a short distance of 5-15 m due to its attraction to visually conspicuous objects [6], [19], [20]. The key emphasis here is on the nearest target concept, which basically implies that factors other than just CO2 alone often cause the mosquito to localize the search at a short distance, as stated in [6], [19], [20]. Non-normalized weights are applied inversely proportional to the distance following this rationale. Note that the community-scale model's form of concentration is consistent with the evidence that larger agglomerates emit stronger odors, thus attracting more mosquitoes [13] (see the illustration in Fig. S9).

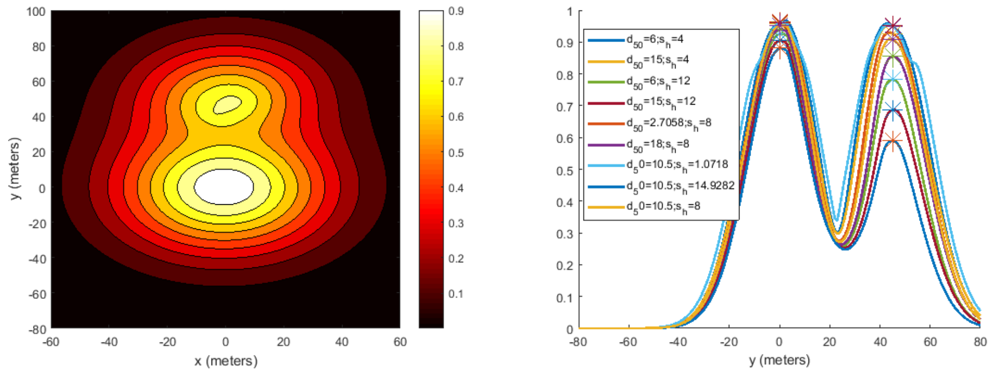

(a) (b)

Figure S9: Softmax function in a special case of two households. The first household includes 6 individuals (located (0,0)) and the other household consists of 2 individuals (located at (0,45)) for different values of d_{50} and s (a) 2D plot, (b) 1D plot along the y axis

Moreso, the increased mosquito greediness, as a result of activation of the heat sensors at a short distance to the host is accounted for by using a linearly distance-dependent scaling factor.The scaling factor's functional behavior results in such a movement that steps in the concentration plume taken towards the host, are always accepted (see Fig. S10a). The design of the algorithm

basically resembles a well-know Simulated Annealing optimization method, introduced in [21]. The difference here is that the 'annealing temperature schedule' is replaced with the 'greediness scale', which is associated to the distance from mosquito to the host. In addition, the scaling factor is further defined in such a way that it depends not only on the distance to the host, but also on the repellent effect. This extension was done to fit the exit rates properly. The scaling factor is computed in the community-level case with distance to the nearest hut, perceived as the nearest visible feature.

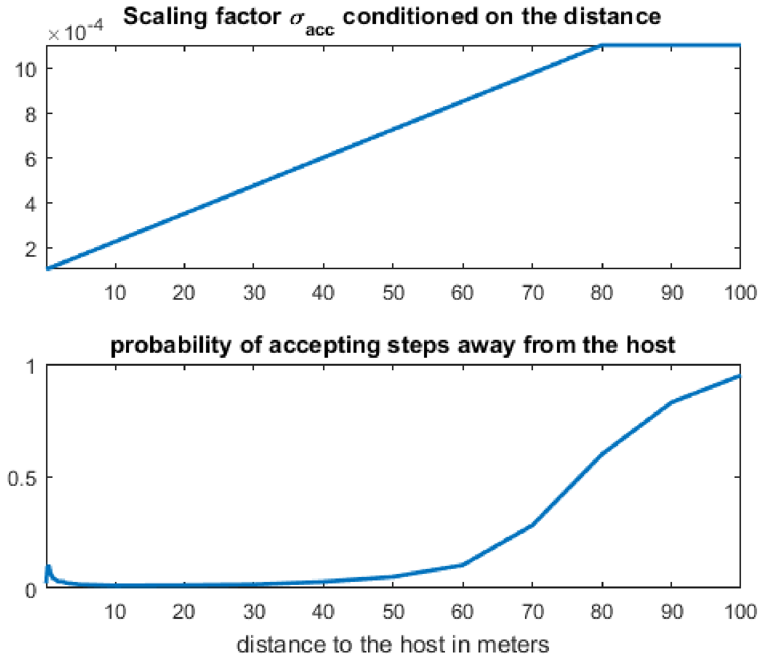

Figure S10: Average probability of accepting candidate steps taken away from the host; as a function of distance from the host.

Mosquito poisoning and mortality model In this work, both natural and insecticide-induced mortality are considered in the model. At the onset, when mosquito has not yet taken the poisonous chemical, the death rate is reduced to the natural mortality. As the dosage of the chemical gradually increases in mosquito, the chemical-induced death occurs from the lethal insecticide dosage. In continuous time, the natural mortality in a declining population is commonly modeled by means of an ordinary differential equation. Here, the continuous-time mortality rate is transformed into probability of death per unit time. This is achieved by discretization in time leading to

agent-based rules rather than the rates. The insecticidal induced mortality is modelled using the total accumulated dosage with effective poisoning impact obtained by a scaling coefficient which depends on the given insecticide used for LLIN treatment. So the total probability of death per unit change in time is modelled as the sum of natural and insecticide-induced mortality.

Repellent model The influence of spatial repellent is imitated by conducting the accept/reject method, with the rejection probability defined by logistic equation. The logistic function is used to describe certain kinds of growth rate that have an S-shaped behaviour. At first, this function grows exponentially, but eventually grow more slowly and levels off, due to certain restrictions. In order to model the repellent effect caused by the net, the function was therefore modified so that the rejection probability at the candidate position attenuates as the distance to the host increases (see Fig. S11).

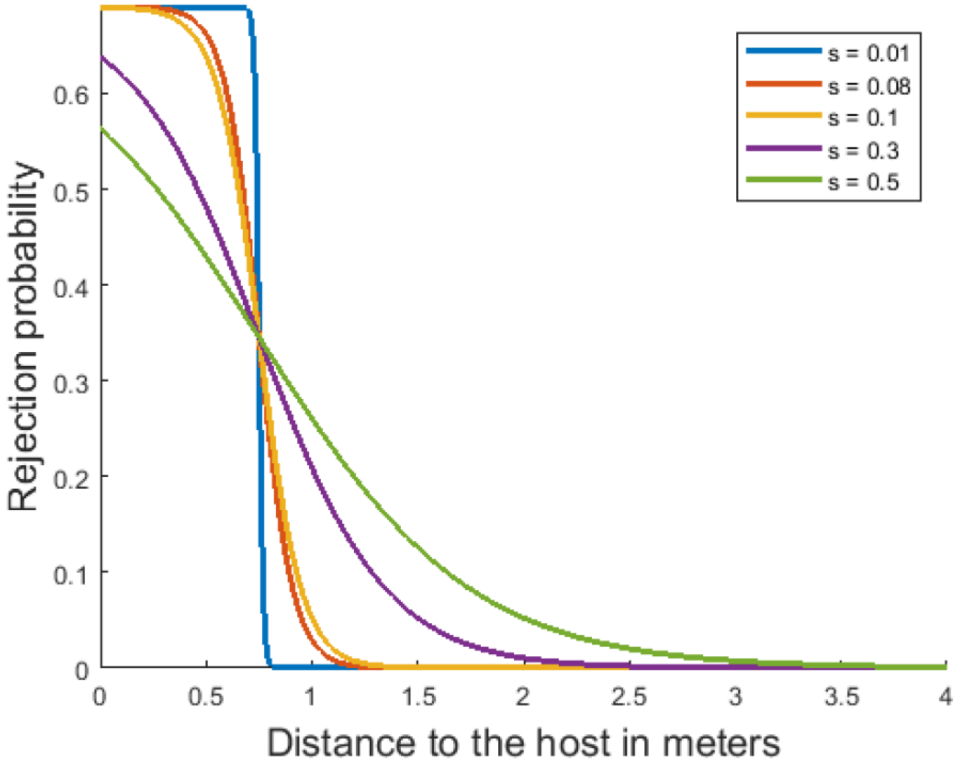

Figure S11: Probability of rejection associated with repellents for different values of the spatial range of repellents.

E.4.2 Emergence

The model behaviour and outputs emerge from the implicit structure of the model.

Hut level In the hut-level case, the impact of LLIN is calibrated by data [1]. Two different model parameterization versions were selected to test various hypotheses explaining the different host-seeking behavior of the species. Both model calibrations gave same overall results for the impact of LLINs. Thus the impact of LLINs is emerging by data and not by the specific hypothesis imposed in the model calibrations (see [11] for more details).

Community level In the community level, the uncertainty from sampled parameters at hut-level is included and a sensitivity analysis with respect to the assumed parameters is conducted using a central composite design. The sensitivity analysis shows that the behavior of the system remains more or less the same with reasonable perturbations in the assumed parameter values (see Fig. S12).

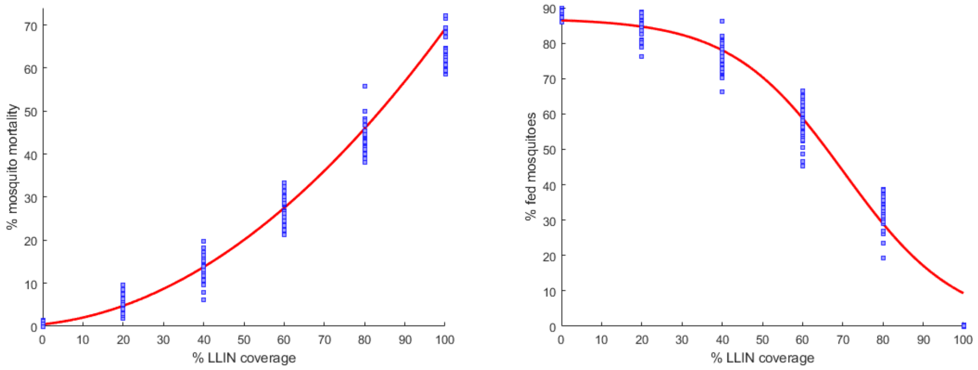

(a) (b)

Figure S12: Uncertainty from the sampled parameters at hut level together with the variability of the community-level assumed parameters for (a) mortality rate (b) fed rate, of *An. gambiae* when confronted with LLIN impregnated with an Alphacypermethrin treatment kit, fitted with respect to partial coverage of LLIN for the household size of 2 when assuming no behavioural alterations caused by the parasite.

E.4.3 Adaptation

In the mosquito host-seeking behavior presented in this work, there is as attractive potential (CO_2 emitted by human) driving the mosquito movement. This makes the mosquito to make more directional movement towards the host and can hardly accept steps away from the host. This potential is given as a solution of the diffusion equation with a point source specified as the Gaussian Kernel centered at a spatial location of the host. This behavior does not change with time and the same set of rules applies regardless of the status of the agent. Additionally, the increased mosquito greediness, as a result of activation of the heat sensors at a short distance to the host is accounted for by adding a linearly distance-dependent scaling factor.

Also, there is a repulsive force that is regarded as *contact irritancy* introduced by the LLINs impregnated with chemicals. The repulsive force can induce early exit from the hut. This effect is generated by a rejection probability of a new position, conditioned on the presence of chemicals. In general, different mosquito behaviors were observed when confronted with each of the chemical treatments under study.

E.4.4 Objectives

There is no individual success or objectives that agents work towards except the general interest to obtain a full blood-meal. They stick to an "indirect objective seeking", in which they simply follow the rules that reproduce observed behavior.

E.4.5 Learning

In the ABM for this study, the navigation capacities are described as the ability to orient in the odour plume emitted from the hosts known as klinotaxis, where mosquito uses its memory of CO_2 concentration from the past to select the next direction of movement. This process is included to enable the mosquito make more directional movement to find the host(s). The mosquitoes do not change their behavior during the course of the simulations.

E.4.6 Prediction

The adaptive behavior of mosquitoes is based on the implicit prediction that, taking steps leading away from the host is likely not to be accepted. This assumption is accurate in the sense that if the new concentration (i.e. in the new mosquito position), is higher than the old concentration, the step is always accepted, otherwise, the step can be accepted with a certain probability. Also, repellency and physical barrier play a role of prediction, as the candidate position is rejected upon being repelled or blocked physically. Mosquitoes do not intentionally make decisions. The 'decisions' are probabilistic and lead to the overall results in a statistical average sense.

E.4.7 Sensing

Motion capacities are captured by considering the mode of movement, switching from a pure random walk in the absence of sensory cues, to a directionally biased random walk, after entering the CO_2 plume. Mosquitoes are usually able to sense the human host only at a distance less than 80 m [12]. To account for a short-distance (less than 3m) behaviour, where increased sensory information induces greater attraction to the host, a third mode of movement is involved. This effect is included by a concentration scaling factor which facilitates more directional movement towards the host. In the community level scenario, the CO_2 concentration sensed by the mosquito at a short distance (less than 15m [6], [19]) is assumed to be the one emitted from the nearest household. The key emphasis here is on the nearest target concept, which basically implies that factors other than just CO2 alone often cause the mosquito to localize the search at a short distance, as stated in [6], [19], [20]. Non-normalized weights are applied inversely proportional to the distance following this rationale, which aligns with the evidence that larger agglomerates emit stronger odors, thus attracting more mosquitoes [13]. The mechanism of sensing (in community-level case) is modelled with the softmax function and the reverse-logistic weights (see Fig. S9). These sensing assumptions included in the simulations are typical for this modelling approach.

E.4.8 Interactions

The ABM presented here consist of non-interactive mosquito agents. However, there is direct interaction between mosquitoes and humans given that mosquitoes can sense and bite the humans.

E.4.9 Stochasticity

The simulation results depends on random numbers, so the output of each experiment is stochastic. Initially, all the mosquito agents occupy randomly generated spatial locations in the simulation domain. In the community-level case, households are randomly located inside the spatial domain. These randomizations used for initialization of spatial positions is done at each successive repetition of the algorithm to average for stochasticity arising from difference in spatial arrangement and position.

The host-seeking process is given by a random walk with accept-reject stepping, with the acceptance probabilities are estimated to match the observed effects associated with mosquito responses to the host, in the presence of the LLIN (such as repulsion and early exit) and poisoning by insecticides. The candidate position is randomly proposed by using uniformly distributed random direction with respect to the previous position. Random numbers are generated from the uniform distribution to compare with the probabilities of accepting (by attraction) and rejecting (associated with repellent) a candidate position, accounting for dead mosquitoes and accounting for the the barriers posed by the net and wall.

In the household-level model, mosquitoes are assumed to randomly choose one of the humans upon entering the household. The scenario is then reduced to the case of a single host in the hut. Moreover, the diversion to other humans which happens after a certain period of time spent in unsuccessful attempts to feed on the protected host was made by choosing another person at random among the other inhabitants of hut. Again, randomization is employed for the multiple biting modelled in the household-level. The maximum number of successful feeding attempts can be up to 5, and this property is randomized and sampled separately for each of the mosquitoes.

In order to ensure statistical accuracy needed for calibration of model parameters, the averaged model outputs obtained by multiple simulations are taken,

using a sufficiently large swarm of mosquitoes in every case. It should also be noted, that the data from [1] are given in percentages, and as such, the absolute number of mosquitoes does not influence the results. However, since the model is stochastic, it is necessary to average all the results over several repetitions. A combination of 6 repetitions and a swarm of 600 mosquitoes (for the hut-level) results in a relatively small variance considering the minimal CPU time. The number of repetitions in the community-level case is larger than in the hut-level experiment, to average for the stochasticity arising from the spatial arrangement of the households. Note that in the community-level simulations, combinations of parameter values are randomly selected from the estimated posteriors at each successive iteration of the algorithm for uncertainty quantification.

E.4.10 Collectives

Collective effects are not included into the model.

E.4.11 Observation

Field data is used to calibrate the hut-level model. At the end of the simulations, the proportion of fed and dead mosquitoes (which are of interest) are recorded, although the proportion of exited mosquitoes can also be recorded. These proportions are recorded separately for two cases: assuming no behavioural alterations and assuming alterations by parasite, separately for the two mosquito species and each of the chemical treatments considered in the study. Also, in case of behavioral alterations, the contact rates are recorded separately for infectious and uninfected mosquitoes. Response surfaces are fitted to the relevant responses (contact and mortality rates) obtained from the ABM simulations, with respect to the household size and the coverage for outputs corresponding to each of the insecticidal treatments, respectively. The response surface is fitted for all the aforementioned cases. The coefficients from the fitted response surfaces can be used when incorporating the ODE-based model of malaria transmission, as they provide the values of the main parameters, which enables the extension of the ABM simulations carried out over a 'snapshot' period of one night to continuous time interval. It was observed that as the coverage with LLINs increases the death rates increase and the fed rates decrease. However, there is insignificant dependence of mortality rates on the household size.

E.5 Initialization

In the hut-level situation, one human agent and with a swarm of 600 mosquitoes is used. At the household-level, one household (with several number of humans) and a swarm of 700 mosquitoes is employed. At the community level, households of different sizes ranging from 2 to 10 people are used. A constant number of 700 mosquitoes and around 20 individuals are used for each experimental run. The mosquitoes can either be infected or uninfected. The number of infectious mosquitoes is constant for a single experiment . This is because, it takes a period of 10 to 12 days for parasites to reach a stage whereby they are ready for transmission whereas the ABM simulation for this study is only for a night. Humans can either be protected or unprotected. The protection is marked with 0 (for unprotected humans) and 1 (for protected humans). The percentage of protected humans for each household remains constant in each of the simulations. Note that for hut-level case, the host is always protected. Again the status of the hosts and the households are marked as not-bitten in the initialization. For each mosquito agent, there is an associated number of states that can be 0 or 1, like dead or alive. For each household, such state is assigned and updated, to track if the mosquito was host-seeking in that household recently. In the simulation model, the mosquitoes are presented as a number of agents in a two-dimensional rectangular domain, initially placed at uniformly generated random spatial locations. In the community-level simulations, mosquitoes are initially randomly positioned inside the experimental domain with multiple households located at a distance not less than 40m from each other, such that there is no competitive attraction caused by vision [6]. Depending on the initial positions of the mosquitoes, the initial concentrations are assigned. The host-seeking time, the number of contacts with the net, and the accumulated dosage of chemicals are initially set to zero for all the mosquitoes and updated at each iteration. In the community-level case, it is assumed that upon entering a new household, the host-seeking time count is reinitialized. This was done to consider the habit of early exit after a certain time spent inside, so-called *exophily*.

E.6 Input data

The model does not use input data to represent time-varying processes.

E.7 Submodels

The equations for the modelled processes in Table S2 are given below:

$$\mathbf{x}^n = \mathbf{x}^{n-1} + \delta\mathbf{W}, \tag{S2}$$

where the increment $\delta\mathbf{W}$ added to $\mathbf{x}^{n-1}$ is sampled in random direction, with a step size given by a normal distribution $N(\mathbf{x}_0, \sigma^2 I)$.

$$C(\mathbf{x}, \mathbf{x^h}) = \exp\left[-\frac{d^2(\mathbf{x}, \mathbf{x}^h)}{2\sigma_a^2}\right], \tag{S3}$$

where $\mathbf{x}$ denotes the position of the mosquito, and C stands for the concentration that enables a mosquito to sense the host at a distance $d(\mathbf{x}, \mathbf{x}^h)$. The standard deviation of the Gaussian σ_a determines a maximal distance at which the mosquito is able to sense the host.

$$\alpha_a(\mathbf{x^n}|\mathbf{x^{n-1}}) = \min\left(1, \frac{p(\mathbf{x^n})}{p(\mathbf{x^{n-1}})}\right), \tag{S4}$$

where $p(\mathbf{x^n})/p(\mathbf{x^{n-1}})$ is the ratio of the attraction potential function $p(\mathbf{x})$ defined at each point $\mathbf{x}$, which depends on the concentration and other attraction factors.

$$\sigma_{acc}(\mathbf{x}, \mathbf{x}^h) = \begin{cases} \sigma_{acc}^1 + \sigma_{acc}^2 d(\mathbf{x}, \mathbf{x}^h), & d(\mathbf{x}, \mathbf{x}^h) \leq 80 \\ \sigma_{acc}^{\max}, & d(\mathbf{x}, \mathbf{x}^h) > 80. \end{cases} \tag{S5}$$

The above function increases from the minimum value of σ_{acc}^1 with a slope given by the parameter σ_{acc}^2 until it is replaced by a constant which suitably provides a purely random movement outside the concentration plume [11].

$$\alpha^{\Delta t} = \min\{1, \mu\Delta t\}, \tag{S6}$$

where $\Delta t = 2$ seconds is used for all simulations, and a value for μ taken from the literature (see [11] for more details).

$$C_{tot}(n+1) = \sum_{i=1}^{n+1} D_i = C_{tot}(n) + D_{n+1}. \tag{S7}$$

where D_i is non-zero in case of hitting the net surface (i.e., equal to the unit dosage), and zero otherwise.

$$C_{tot}(n+1) = C_{tot}(n) + D_{n+1} - \alpha C_{tot}(n)\Delta t. \tag{S8}$$

$$\alpha_p^{\Delta t}(n) = \mu_p C_{tot}(n)\Delta t, \tag{S9}$$

where the effective poisoning impact is obtained by a scaling coefficient μ_p which depends on the given insecticide used for LLIN treatment.

$$C_{rej} = r\left[1 - 1/\Big(1 + \exp\big(-\big(d(\mathbf{x}, \mathbf{x}^h) - d_{50}\big)/s\big)\Big)\right], \tag{S10}$$

where $d(\mathbf{x}, \mathbf{x}^h)$ denotes the distance from the mosquito to the protected human and r ranges from 0 to 1. The parameters d_{50} and s determine the range of coverage and the spread of the chemical. The logistic function is modified such that the rejection probability at the candidate position $\mathbf{x}$ amplifies as the mosquito approaches the source of repellent.

$$\sigma_{acc}(\mathbf{x}, C_{tot}) = \sigma_{acc}(\mathbf{x}) + \mu_e \cdot C_{tot}, \tag{S11}$$

where C_{tot} denotes the total dosage of chemical consumed by the mosquito (see Equation S7).

References

[1] Kitau J, Oxborough RM, Tungu PK, Matowo J, Malima RC, et al. Species shifts in the *Anopheles gambiae* complex: do LLINs successfully control *Anopheles arabiensis*?. PLoS One. 2012;7:e31481.

[2] Haario H, Laine M, Mira A, Saksman E. DRAM: efficient adaptive MCMC. Stat Comput. 2006;16:339-54.

[3] Wallace DI, Southworth BS, Shi X, Chipman JW, Githeko AK. A comparison of five malaria transmission models: benchmark tests and implications for disease control. Malar J. 2014;13:268.

[4] Okumu FO, Mbeyela E, Lingamba G, Moore J, Ntamatungiro AJ, Kavishe DR, et al. Comparative field evaluation of combinations of long-lasting insecticide treated nets and indoor residual spraying, relative to either method alone, for malaria prevention in an area where the main vector is *Anopheles arabiensis.* Parasit Vectors, 2013,6:46.

[5] World Health Organization. Guidelines for testing mosquito adulticides for indoor residual spraying and treatment of mosquito nets. 2006.

[6] Bidlingmayer WL, Hem DG. The range of visual attraction and the effect of competitive visual attractants upon mosquito (*Diptera: Culicidae*) flight. Bull Entomol Res. 1980;70:321-42.

[7] Vickers NJ. Mechanisms of animal navigation in odour plumes. Biol Bull. 2000;198:203-12.

[8] Jones J, Murray GD, McCall PJ. A minimal 3D model of mosquito flight behavior around the human baited bed net. Malar J. 2021;20:24.

[9] Nardini L, Christian RN, Coetzer N, Ranson H, Coetzee M, Koekemoer LL. Detoxification enzymes associated with insecticide resistance in laboratory strains of *Anopheles arabiensis* of different geographic origin. Parasit Vectors. 2012;5:113.

[10] Kerkut GA, Gilbert LI. Comprehensive Insect Physiology, Biochemistry and Pharmacology. Oxford: Pergamon; 1985.

[11] Shcherbacheva A, Killeen GF, Haario H. Modelling host-seeking behaviour of African malaria vector mosquitoes in the presence of long-lasting insecticidal nets.Math Biosci. 2018; 295:36-47.

[12] Cardé RT. Odour plumes and odour-mediated flight in insects. Ciba Found Symp. 1996;200:54-66; discussion 66-70.

[13] Cummins B, Cortez R, Foppa IM, Walbeck J, Hyman JM. A spatial model of mosquito host-seeking behaviour. PLoS Comput Biol. 2012;8:e1002500.

[14] Tang W, Bennett DA. Agent-based modeling of animal movement: a review. Geogr Compass. 2010;4:682-700.

[15] Metropolis N, Rosenbluth AW, Rosenbluth MN, Teller AH, Teller E. Equation of state calculations by fast computing machines. J Chem Phys. 1953;21:1087-92.

[16] Crank J. The mathematics of diffusion. Oxford university press; 1979.

[17] Bishop CM. Pattern Recognition and Machine Learning. Springer; 2006.

[18] Montague PR. Reinforcement Learning: An Introduction, by Sutton, RS and Barto, AG. Trends Cogn Sci. 1999;3:360.

[19] Hawkes FM, Dabiré RK, Sawadogo SP, Torr SJ, Gibson G. Exploiting *Anopheles* responses to thermal, odour and visual stimuli to improve surveillance and control of malaria. Sci Rep. 2017;7:17283.

[20] van Breugel F, Riffell J, Fairhall A, Dickinson MH. Mosquitoes use vision to associate odour plumes with thermal targets. Curr Biol. 2015;25:2123-9.

[21] Kirkpatrick S, Gelatt CD, Vecchi MP. Optimization by simulated annealing. Science. 1983;220:671-80.
